# Adolescent Depressive Symptom Trajectories From Before to After the COVID-19 Pandemic

**DOI:** 10.1001/jamanetworkopen.2025.45987

**Published:** 2025-12-01

**Authors:** Mārtiņš M. Gataviņš, Kate T. Tran, Elina Visoki, Tyler M. Moore, Kevin W. Hoffman, Tal Shilton, Laura M. Schultz, Laura Almasy, Anthony D. Mancini, Ran Barzilay

**Affiliations:** 1 Department of Child and Adolescent Psychiatry and Behavioral Sciences, Children’s Hospital of Philadelphia, Philadelphia, Pennsylvania; 2 Lifespan Brain Institute of Children’s Hospital of Philadelphia and Penn Medicine, Philadelphia, Pennsylvania; 3 Department of Psychiatry, Perelman School of Medicine, University of Pennsylvania, Philadelphia; 4 Child-Adolescent Psychiatry Division, Sheba Medical Center, Ramat Gan, Israel; 5 Sackler School of Medicine, Tel Aviv University, Tel Aviv, Israel; 6 Department of Biomedical and Health Informatics, CHOP, Philadelphia, Pennsylvania; 7 Department of Genetics, Perelman School of Medicine, University of Pennsylvania, Philadelphia; 8 Department of Psychology, Pace University, Pleasantville, New York

## Abstract

**Importance:**

Adolescent depression rates increased during the COVID-19 pandemic globally. Data on risk and resilience factors can inform prevention and intervention strategies during a major adversity.

**Objective:**

To characterize depression symptom trajectories from before, during, and after the lockdown stages of the pandemic and to identify prospective risk and resilience factors.

**Design, Setting, and Participants:**

This cohort study followed participants from the Adolescent Brain Cognitive Development Study ascertained through 21 sites across the US from before March 2020 to February 2022. The design and hypotheses were preregistered in March 2024. Analyses were conducted from April to November 2024.

**Exposures:**

Prepandemic risk and resilience factors spanning developmental stage, household environment, peer relationships, and depression polygenic risk.

**Main Outcomes and Measures:**

The primary outcome was depression-susceptible symptom trajectory of low prepandemic and high postpandemic symptoms. Trajectories of depression symptom score (6 symptoms: depressed mood, anhedonia, guilt or worthlessness, fatigue, sleep, and concentration impairments) were modeled using logistic regression models with prepandemic factors (independent variables) explaining trajectory class (dependent variable; resilient, 0; depression susceptible, 1), adjusting for sex and age at the pandemic onset.

**Results:**

In the sample of 3512 participants included in the analyses (mean [SD] age in March 2020, 12.05 [0.85] years; 1672 [47.6%] female), a 3-class trajectory model identified resilient (low symptoms throughout follow-up, 3027 participants [86.2%]), depression-susceptible (low symptoms before the pandemic and high symptoms after the pandemic, 326 participants [9.3%]), and chronically high (159 participants [4.5%]) symptom trajectory classes. Girls were overrepresented in the depression-susceptible vs the resilient group (240 girls [73.6%] vs 1327 girls [43.8%]). The depression-susceptible trajectory, compared with the resilient trajectory, was associated with late pubertal or postpubertal stage before the pandemic (odds ratio [OR], 1.46; 95% CI, 1.08-1.96; *P* = .01), prepandemic family conflict (OR, 1.23; 95% CI, 1.10-1.38; *P* < .001), peer bullying (OR, 1.71; 95% CI, 1.11-2.65; *P* = .02), cyberbullying (OR, 2.28; 95% CI, 1.45-3.59; *P* < .001), maternal history of depression (OR, 1.52; 95% CI, 1.16-1.99; *P* = .002), polyenvironmental adversity exposure (OR, 1.28; 95% CI, 1.13-1.45; *P* < .001), and polygenic risk of depression (OR, 1.28; 95% CI, 1.03-1.59; *P* = .02 among those of European-like genetic ancestry, but not those of African-like ancestry). Greater prepandemic parental monitoring (OR, 0.81; 95% CI, 0.73-0.91; *P* < .001) and problem-solving skills (OR, 0.80; 95% CI, 0.66-0.97; *P* = .02) were associated with lower depression susceptibility.

**Conclusions and Relevance:**

In this cohort study of adolescent depression trajectories throughout COVID-19, girls, teens in later puberty, and those with experiences of peer bullying, cyberbullying, and greater family conflict before the pandemic were more susceptible to developing depressive symptoms that persisted after the pandemic, whereas prepandemic parental monitoring and problem-solving skills were identified as prospective modifiable resilience factors.

## Key Points

**Question:**

How did adolescent depressive symptoms change from before to after the COVID-19 pandemic, and which prepandemic factors were associated with symptom trajectories?

**Findings:**

This cohort study of 3512 adolescents identified that 86.2% were resilient and 9.3% were depression susceptible, with greater susceptibility in girls and late-pubertal youth. Prepandemic factors associated with depression susceptibility included bullying, family conflict, maternal history of depression, and polygenic risk; factors associated with resilience included parental monitoring and problem-solving skills.

**Meaning:**

These findings suggest that most adolescents maintained stable mental health from before to after the COVID-19 pandemic, with diverse prepandemic environmental, psychological, and genetic factors jointly shaping individual risk and resilience trajectories.

## Teaser

This cohort study examines depression symptom trajectories from before, during, and after the lockdown stages of the COVID-19 pandemic and identifies factors associated with risk and resilience among US adolescents.

## Introduction

The global surge in adolescent depression observed during the COVID-19 pandemic has raised widespread concerns for youth mental health.^1,2,3^ The ripple effects of the pandemic and its associated policy and social changes on adolescents’ lives have been pervasive.^4,5^ Studies suggest that there are multiple pandemic-related contributors to poorer adolescent mental health, including financial strain,^6^ social isolation,^2,7,8^ and family conflict.^9^

Resilience is the capacity to withstand adversity while retaining stable mental health.^10,11,12^ A resilient outcome after the COVID-19 pandemic can be defined as maintaining low symptom burden throughout the pandemic. Prior research on depression trajectories following adversity suggests that a resilient outcome is the most common.^13,14,15,16^ Despite results highlighting a substantial increase in depression and anxiety during the pandemic,^2,3^ patterns of resilience have also been observed early in the pandemic,^17^ whereby more individuals exhibited low symptom (resilient) trajectories than symptom-increase trajectories (susceptible) during lockdown stages.^2,18,19^ Limited research has examined mental health trajectories during the later pandemic stage, after vaccines were made available.^20^

Resilience is a multidimensional construct.^10,11,12^ Prepandemic research suggests that several resilience factors, including neurocognitive (eg, inhibitory control^21^), environmental (eg, parenting styles^22^ and adversity^23^), and genetic factors^24,25,26^ contribute to resilient outcomes.^10,12,22^ Studies suggest that multiple factors influenced adolescent mental health during the COVID-19 pandemic, including public health restrictions and individual socioeconomic factors.^20,27,28^ Less is known on how prepandemic individual-level risk and resilience factors contributed to interindividual differences in depressive symptom trajectory throughout the pandemic. A broader understanding of these prospective risk and resilience factors could improve depression risk classification and screening among a generation who lived through the pandemic as adolescents and can inform development of interventions that promote youth’s resilience in future public health emergencies.^29,30^

The Adolescent Brain Cognitive Development (ABCD) Study tracks a large cohort of American youth ascertained between 2016 and 2018 and followed annually from late childhood into adolescence, offering a unique opportunity to examine mental health trajectories from before to after the pandemic. Although prior ABCD Study analyses identified risk and protective factors during the pandemic,^20,31,32^ the role of individual-level prepandemic factors in shaping depression trajectories after the pandemic remains unclear. To address this gap, we used the ABCD Study as a natural experiment to identify childhood variables associated with increased susceptibility or resilience to depressive symptoms during a period that spanned the pandemic. We hypothesized that, consistent with prepandemic literature,^10,33,34,35^ most youths would exhibit a resilient trajectory and that multidimensional risk and resilience factors, including biological (pubertal status and polygenic risk), intrapersonal (eg, problem solving), interpersonal (eg, family and peer relationships), and socioeconomic factors (eg, household income), would prospectively contribute to variability in depressive symptoms trajectories.

## Methods

### Participants

This cohort study included data from the ABCD Study (Release 5.1), which follows a cohort of US children recruited at ages 9 to 10 years in 2016 to 2018 from 21 US sites, with annual assessments through adolescence.^36^ In addition to the annual assessments (ABCD main study), during the COVID-19 pandemic, 7 surveys were administered from May 2020 to July 2021 (ABCD COVID substudy).^37^ For this study, we included participants who were assessed at 3 time periods: before the pandemic (latest ABCD main study assessment before March 2020, termed *prepandemic*), during the pandemic (at least 1 assessment between March 2020 and July 2021), and after the lockdown period (ABCD main study assessment conducted after July 2021, up to February 2022, termed *postpandemic*). Participants provided assent, and parents or caregivers provided written informed consent. ABCD Study protocol was approved by the University of California, San Diego institutional review board and was exempted from a full review by the Children’s Hospital of Philadelphia institutional review board. This study followed the Strengthening the Reporting of Observational Studies in Epidemiology (STROBE) reporting guidelines.

### Assessment of Depressive Symptoms

To harmonize measurement of depressive symptoms across the main ABCD Study (assessed with Kiddie Schedule for Affective Disorders and Schizophrenia [K-SADS],^38,39^ Brief Problem Monitor,^40^ and Munich Chronotype Questionnaire^41,42^) and COVID substudy (assessed with National Institutes of Health Emotion Toolbox Sadness^6,20,31,43,44^ and Positive Affect^32^ Scales, and abbreviated Munich Chronotype Questionnaire^31,41,42^), 3 child-adolescent psychiatrists (K.W.H., T.S., and R.B.) reached a consensus on criteria for 6 depressive symptoms, according to *Diagnostic and Statistical Manual of Mental Disorders* (Fifth Edition) (*DSM-5*) criteria, assessed in both studies: depressed mood, anhedonia, fatigue, sense of guilt or worthlessness, sleep, and concentration problems. Thereafter, a score of 0 to 6 endorsed symptoms was derived for each participant at each time point. eAppendix 1 and eTables 1 to 3 in Supplement 1 detail calculation of symptom score and handling of missing data.

### Prepandemic Risk and Resilience Factors

Risk and resilience factors (detailed in eAppendix 2 in Supplement 1) were selected emphasizing multilevel developmental influences.^45^ These measures spanned biological and psychosocial factors, including self-reported pubertal stage derived from a sum of 3 questions about pubertal milestones,^46,47^ major depression polygenic risk score (hereafter, *polygenic risk*) calculated using posterior genome-wide association studies for European^48^ and African ancestry^49^ (eAppendix 2 in Supplement 1), household income, home environment (self-report family conflict^50,51,52^ and parental monitoring^53,54^), interpersonal stressors (self-reported experiences of cyberbullying and peer bullying^55,56,57^), problem-solving skills and prosocial behaviors (assessed using self-report 6-item Wills Problem Solving Scale^58,59^ and 3-item Prosocial Behavior Survey^60,61,62^), and caregiver-reported family history of depression. We also examined structural environment (geocoded Area Deprivation Index) and an aggregate polyenvironmental (exposomic) measure of prepandemic adversity that we previously derived using dimensionality reduction of 348 exposure variables (eAppendix 2 in Supplement 1).^63^

### Statistical Analysis

**Figure 1.**
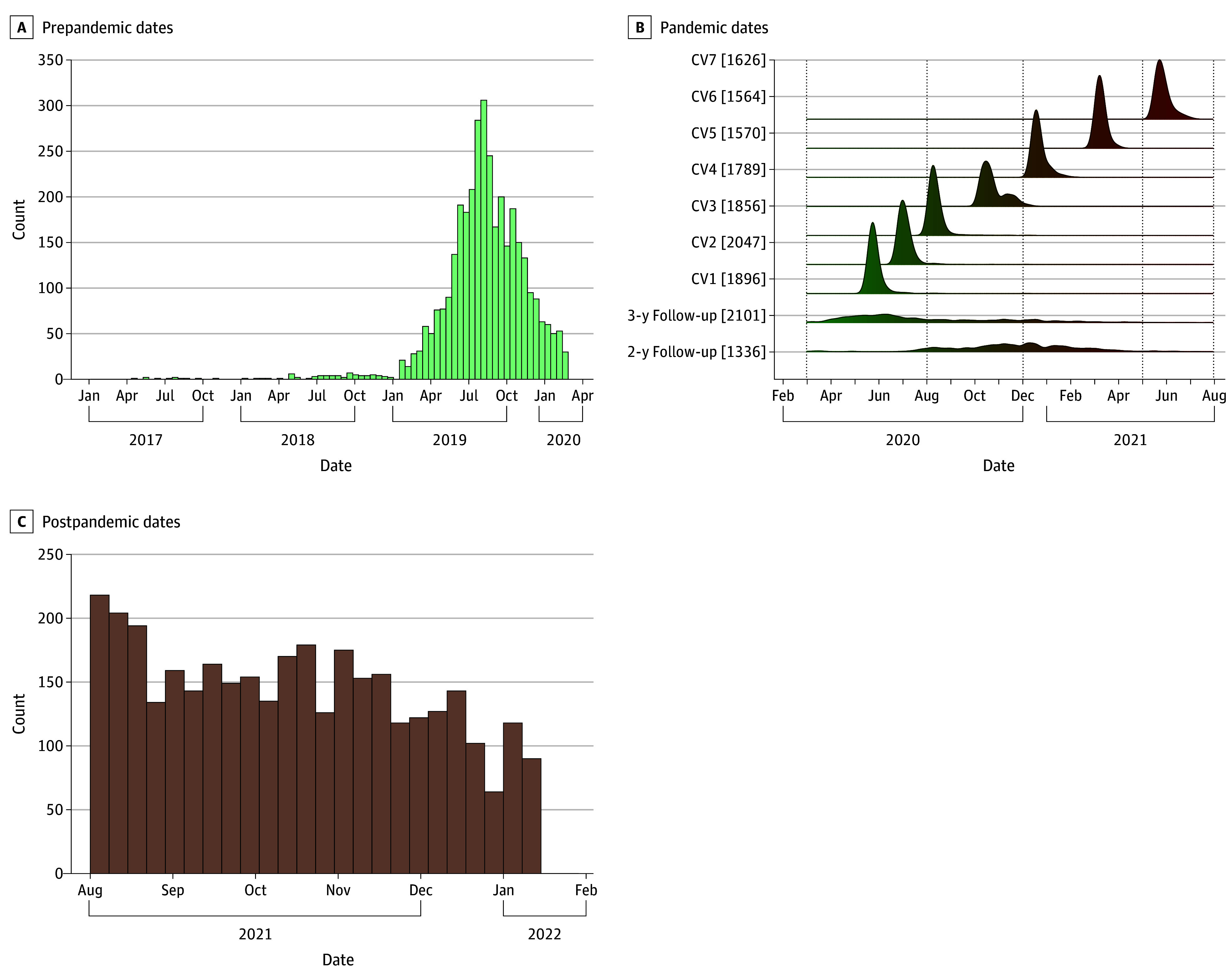
Data Collection Dates and Correspondence to Adolescent Brain Cognitive Development Study Collection Events A, Distribution of prepandemic collection dates of Adolescent Brain Cognitive Development (ABCD) main study data for all included participants (n = 3512). Each histogram bar denotes a 2-week period of collection. B, Distribution of data collection dates for all included participants (n = 3512) during the pandemic, including assessment waves of the COVID-19 substudy (using the National Institutes of Health Emotion Toolbox and abbreviated Munich Chronotype Questionnaire [MCTQ] for depression symptom assessment) and ABCD main study (using Kiddie Schedule for Affective Disorders and Schizophrenia [K-SADS], Brief Problem Monitor, and MCTQ). The number of total participants with an assessment in that wave (each wave is denoted with CV and a number) is denoted in the brackets. C, Distribution of postpandemic ABCD main study collection dates for all participants (n = 3512). Each histogram bar denotes a 2-week period of data collection. Dates of events corresponding to collection dates is available in eFigure 1 in Supplement 1.

We calculated depression scores for all participants included in the study and organized them into 6 time bins (Figure 1; eFigure 1 and eAppendix 1 in Supplement 1). These were then used to identify subpopulations with different trajectories (further termed *classes*) of depressive symptoms in all selected participants using latent growth mixture modeling. Then, we focused on participants in trajectory classes most consistent with resilience (low or constant) and depression susceptibility (increasing). We first performed univariable comparisons between the 2 classes, followed by multivariable analyses testing associations of prepandemic measures as independent variables and trajectory class (depression susceptible vs resilient) as the outcome. The analysis plan and hypotheses were preregistered in March 2024.^64^ Analyses were conducted between April and November 2024 using R statistical software version 4.4.1 (R Project for Statistical Computing).^65^ A 2-tailed *P* < .05 was considered statistically significant.

#### Trajectory Modeling of Depressive Symptoms From Before to After the COVID-19 Pandemic

We applied latent growth mixture modeling to depression symptom scores and compared model fit solutions with 1 to 5 classes.^66^ Within each class, the intercept growth factor variance was not constrained, allowing for individual heterogeneity in baseline depression symptoms, while the slope variance was fixed to zero.^67^ Trajectory modeling was conducted in Mplus version 8^68^ on all available data using a robust full information maximum likelihood estimation procedure for handling partially missing (ie, ≥1 time points) depression symptom scores, assuming random missingness.^69^ We selected the best model by balancing multiple criteria: Bayes and Akaike information criteria assessing model fit, Lo-Mendell-Rubin adjusted likelihood ratio test to evaluate whether addition of another class significantly improved the solution,^70^ entropy values to gauge class separability,^71^ and overall interpretability.^72,73^

Because ABCD Study data are nested (with participants grouped in families and sites), we fit 2-level models, clustering (ie, modeling the random effects) individual participants (level 1) by family (level 2) and then stratifying by site, a correction of SE differences, producing robust, site-controlled outputs that comprehensively account for the hierarchical structure of ABCD Study. This approach was preferred over 3-level models that are often computationally prohibitive and may not converge in practice.^74^

#### Univariable Comparison of Resilient vs Depression-Susceptible Trajectory Classes

We compared demographics between the 2 trajectory classes of interest, depression susceptible and resilient, using *t* tests for continuous variables, χ^2^ tests for continuous variables, and Fisher exact tests for variables with sparse frequency tables.^75^ To assess internal validity of the depression-susceptible class, we compared rates of participants meeting clinically meaningful diagnostic criteria of depression, as well as other diagnoses, using K-SADS assessment.

When comparing all 3 classes, we used 1-way analysis of variance for continuous variables and the Pearson χ^2^ test for categorical variables. For pairwise post hoc testing, we used the Tukey honestly significant difference test for continuous variables and post hoc pairwise Pearson χ^2^ or Fisher exact tests for categorical variables.

#### Multivariable Logistic Mixed-Effects Regression Models

To test associations of prepandemic measures with trajectory classes, we used logistic mixed-effects regression models (mixed models) with a binary dependent variable denoting risk (resilient, 0; depression susceptible, 1) and individual prepandemic measures as independent variables. Models adjusted for age in March 2020, sex assigned at birth, and prepandemic depressive symptom score, consistent with previous literature on age and sex differences in psychopathology in ABCD^62^ and with our aim to delineate environmental, individual, and biological risk and resilience factors. To account for random effects, mixed models were nested in 2 levels, according to family-relatedness and then study site, and included random intercepts for site and family. We used listwise deletion for missing data.

We analyzed combined polygenic and exposomic associations, and their interactions, with trajectory class. Models including polygenic risk were run separately for European-like and African-like genetic ancestries, first without and then with interaction terms.

#### Sensitivity Analyses

To address the possible influence of our definition of depressive symptoms, we tested trajectory models using broader (more sensitive, less specific) symptom definitions (eTable 2 in Supplement 1). To address the possibility that our findings were associated with data missingness, we reran trajectory models using scores from imputed data (eAppendix 1 in Supplement 1). To address interindividual differences in experiences during the pandemic, we added analyses that tested associations of prepandemic risk and resilience factors with depressive symptoms trajectories while adjusting for 2 established pandemic-related stressors: financial strain and family conflict during the pandemic.^6^

## Results

### Trajectories of Adolescents’ Depressive Symptoms From Before to After the COVID-19 Pandemic

**Figure 2.**
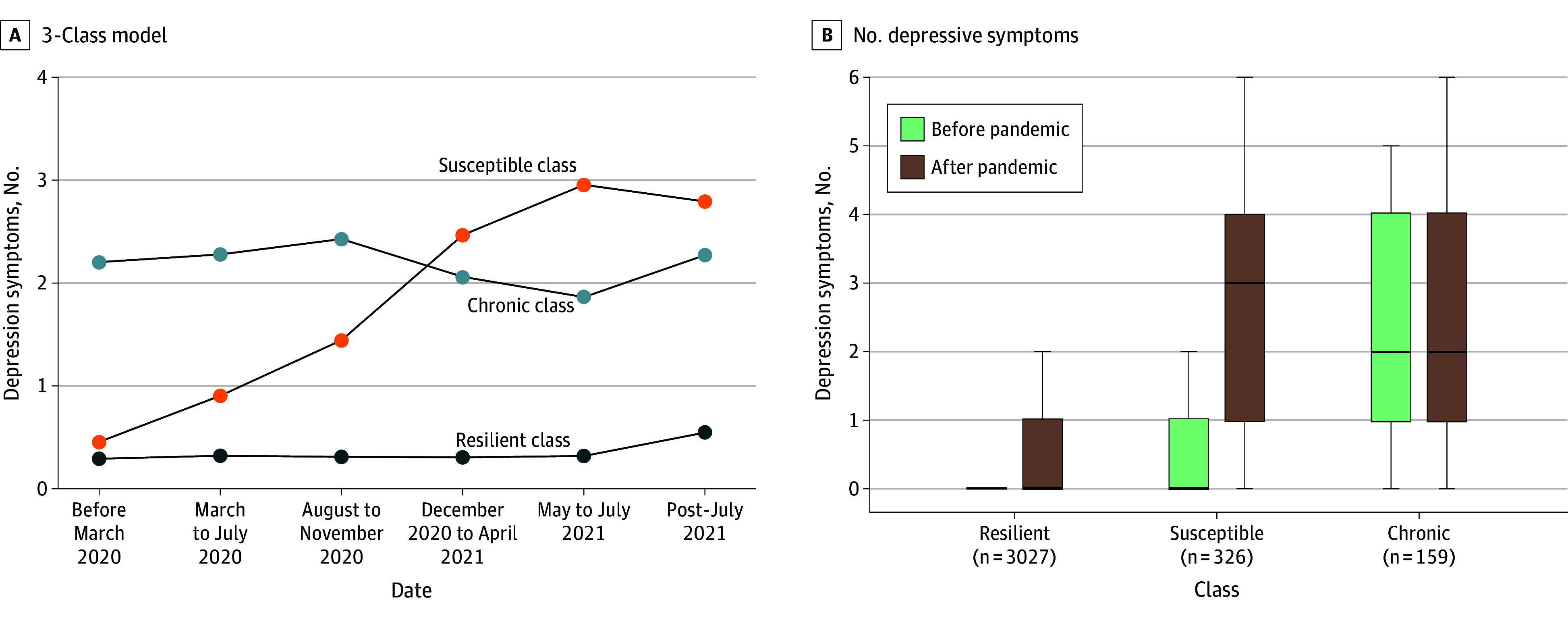
Depression Symptom Trajectory Model Solution and Depressive Symptoms Before and After the Pandemic A, A 3-class solution from latent growth mixture models of depressive symptom scores. The 3 class trajectories were distributed into a majority resilient class (3027 participants [86.2%]), a smaller depression-susceptible class (326 participants [9.3%]), and the smallest chronic class (159 participants [4.5%]). B, Prepandemic and postpandemic number of depressive symptoms, where the box midline and upper and lower limits denote median and first and third quartiles, respectively, and whiskers denote the minimum and maximum.

We identified 3512 youths with prepandemic, during-pandemic, and postpandemic assessments (mean [SD] age in March 2020, 12.05 [0.85] years; 1672 [47.6%] female; from 2967 families and 21 sites) (Figure 1). A 3-class model provided the best solution of depressive symptom trajectories based on fit statistics and theoretical interpretability (Figure 2A; eTable 4 in Supplement 1). As hypothesized, most youths showed a stable, nonsignificant trajectory of low (near-zero) depressive symptoms (resilient, 3027 participants [86.2%]). The remaining youths belonged either to a chronic high-symptom trajectory class (chronic, 159 participants [4.5%]), or a class with low prepandemic depressive symptom burden and an increasing slope (depression susceptible, 326 participants [9.3%]). Only the depression-susceptible class showed a significant trajectory slope (β [SE], 0.54 [0.08]; *P = *.002) (eTable 5 in Supplement 1). Adolescents in the depression-susceptible class reported a median (IQR) of 3 (1-4) depressive symptoms after the pandemic, up from a median (IQR) of 0 (0-1) before the pandemic. In contrast, resilient youths maintained low symptom levels, with a median (IQR) of 0 (0) before the pandemic and 0 (0-1) after the pandemic (Figure 2B). Notably, 123 youths (56.2%) in the depression-susceptible class met *DSM-5* criteria for major depression after the pandemic, as determined by the K-SADS–derived depression diagnosis. Depression-susceptible youths were more likely to meet criteria for other psychiatric diagnoses after the pandemic (eTable 6 in Supplement 1).

### Comparison of Resilient With Depression-Susceptible Youths

The Table details demographic comparisons by trajectory class between resilient and depression-susceptible youths (3353 participants). Compared with resilient youths, depression-susceptible youths were more likely to be female (240 girls [73.6%] vs 1327 girls [43.8%]) and were slightly but statistically significantly older (mean [SD] age, 12.19 [0.82] vs 12.03 [0.86] years), and their parents tended to have more years of education. Comparison of all 3 classes (resilient, depression susceptible, and chronic) is detailed in eTable 7 in Supplement 1.

**Table.** Demographic Comparisons of Resilient and Depression-Susceptible Youths

Characteristic	Participants, No. (%)^a^		*P* value^b^
	Resilient (n = 3027)	Depression susceptible (n = 326)	
Sex			
Female	1327 (43.8)	240 (73.6)	<.001
Male	1700 (56.2)	86 (26.4)	
Race and ethnicity^c^			
American Indian or Alaska Native	95 (3.1)	12 (3.7)	.60
Asian	200 (6.6)	31 (9.5)	.05
Black	681 (22.5)	75 (23.0)	.80
Hispanic	586 (19.6)	74 (22.8)	.20
Native Hawaiian or Other Pacific Islander	15 (0.5)	2 (0.6)	.70
White	2212 (73.1)	235 (72.1)	.70
Multiracial	356 (11.8)	45 (13.8)	.30
Other	187 (6.2)	14 (4.3)	.20
Household income below federal poverty line	397 (14.5)	50 (16.8)	.30
Age in March 2020, mean (SD), y	12.03 (0.86)	12.19 (0.82)	<.001
Born in US	2923 (96.8)	314 (96.6)	.90
Parent education			
Below high school	160 (5.3)	14 (4.3)	.049
High school graduate	279 (9.2)	34 (10.5)	
Some post–high school education	718 (23.7)	99 (30.6)	
Bachelor’s degree	759 (25.1)	77 (23.8)	
Master’s degree or above	1109 (36.7)	100 (30.9)	
Annual household income, $			
<25 000	350 (12.5)	41 (13.4)	.09
25 000 to <50 000	380 (13.6)	45 (14.7)	
50 000 to <100 000	705 (25.2)	93 (30.4)	
100 000 to <200 000	940 (33.6)	95 (31.1)	
≥200 000	423 (15.1)	32 (10.5)	

^a^ Percentages are calculated from the number of nonmissing values (missingness of all measures is <8%).

^b^ For continuous variables, Welch 2-sample *t* test was used. For categorical variables, either Pearson χ^2^ or Fisher exact test was used (if either group had a count <5, the latter was used).

^c^ Race and ethnicity were caregiver-reported from the survey options for ethnicity (Hispanic/not Hispanic) and race: American Indian or Alaska Native, Asian, Black, Hispanic, multiracial, Native Hawaiian or Other Pacific Islander, White, or other (not specified).

To assess biological relevance of age differences between classes, we compared prepandemic pubertal stages. The depression-susceptible class had significantly more youths at late or postpubertal stages than the resilient class (79 youths [25.7%] vs 360 youths [12.6%]) (eFigure 2 in Supplement 1). Multivariable models revealed that compared with their peers in early puberty, when controlling for sex, youths in late puberty or postpuberty before the pandemic had greater odds of belonging to the depression-susceptible class (odds ratio [OR], 1.46; 95% CI, 1.08-1.96; *P* = .01) (eTable 8 in Supplement 1). There was no significant interaction between sex and pubertal status (*P* for interaction = .52). Results remained unchanged when using the continuous pubertal development score (eTable 9 in Supplement 1).

To disentangle the associations of sex and pubertal status with depression susceptibility, we performed separate trajectory analyses for youths who were prepubertal and those who were late pubertal or postpubertal throughout the study (eFigure 2 and eTable 10 in Supplement 1). The late pubertal or postpubertal group mirrored the main findings, with clear resilient, depression-susceptible, and chronic classes; by contrast, the prepubertal group showed a smaller increase in symptoms within the depression-susceptible class, suggesting that depression susceptibility was more pronounced among youths who entered the pandemic already in late puberty or postpuberty.

### Prepandemic Risk and Resilience Factors

**Figure 3.**
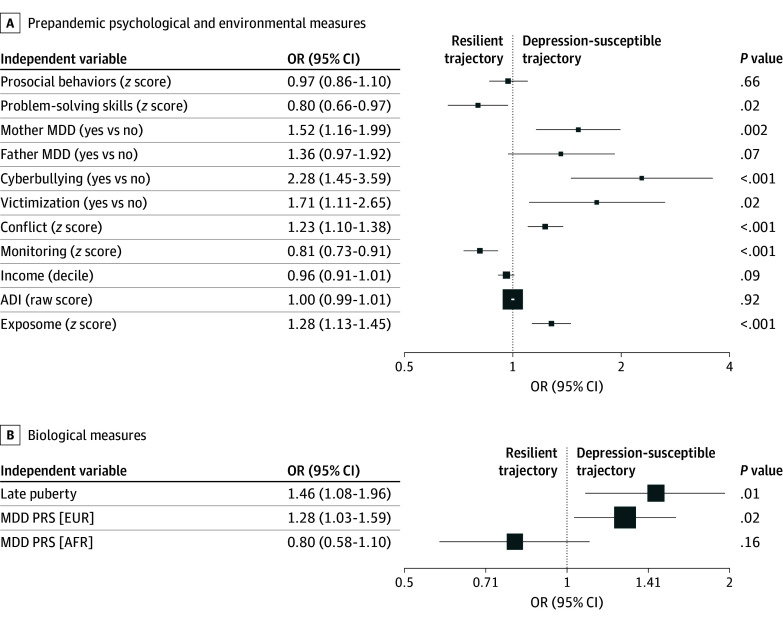
Multilevel Prospectively Measured Prepandemic Risk and Resilience Factors We performed logistic mixed effects regression models using binary outcome measures (resilient = 0 and depression-susceptible = 1) and a set of independent variables from prepandemic assessments. Odds ratios (ORs) greater than 1 indicate that the factor is associated with a depression-susceptible trajectory; an OR less than 1 indicates the factor is associated with a resilient trajectory. A, Prepandemic psychological and environmental measures. B, Biological measures. OR axis is plotted using log_10_ scaling. ADI indicates Area Deprivation Index; AFR, African-like genetic ancestry; EUR, European-like genetic ancestry; MDD, major depressive disorder; and PRS, depression polygenic risk score.

Figure 3 summarizes associations of prepandemic factors with depression susceptibility compared with the resilient class. Youths were more resilient (ie, OR <1 of belonging to depression-susceptible class) if they had better prepandemic problem-solving skills (OR, 0.80; 95% CI, 0.66-0.97; *P* = .02) (eTable 11 in Supplement 1) or more prepandemic parental monitoring (OR, 0.81; 95% CI, 0.73-0.91; *P* < .001) (eTable 12 in Supplement 1). Youths were more depression susceptible, as measured by binary variables, if they experienced greater prepandemic cyberbullying (OR, 2.28; 95% CI, 1.45-3.59; *P* < .001), prepandemic peer bullying (OR, 1.71; 95% CI, 1.11-2.65; *P* = .02) (eTable 13 in Supplement 1), or had maternal family history of depression (OR, 1.52; 95% CI, 1.16-1.99; *P* = .002) (eTable 14 in Supplement 1). From continuous variables, participants from homes with greater prepandemic family conflict were more likely to be depression susceptible (OR, 1.23; 95% CI, 1.10-1.38; *P* < .001) (eTable 12 in Supplement 1). No significant associations with class membership were observed for prepandemic prosocial behaviors (eTable 11 in Supplement 1), household income (eTable 15 in Supplement 1), or neighborhood deprivation.

### Exposomic and Polygenic Associations

Greater prepandemic adverse exposome was associated with depression susceptibility (OR, 1.28; 95% CI, 1.13-1.45; *P* < .001) (eTable 15 in Supplement 1). Polygenic risk of depression was associated with depression susceptibility in the 1525 participants of European ancestry (OR, 1.28; 95% CI, 1.03-1.59; *P* = .02) (Figure 3; eTable 16 in Supplement 1). Analyses in the 466 participants of African ancestry did not reveal significant associations of polygenic risk with depression susceptibility (eTable 16 in Supplement 1).

**Figure 4.**
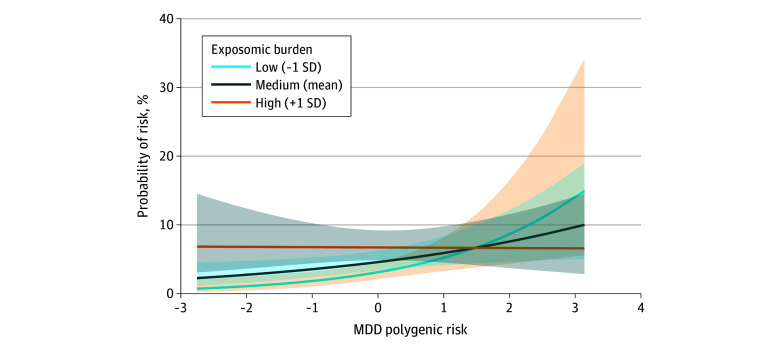
Interaction Between Polygenic Risk and Polyenvironmental Adversity in European-Like Ancestry Participants We performed logistic mixed effects regression models using binary dependent variables (resilient = 0 and depression-susceptible = 1) and an interaction term of prepandemic exposome score and polygenic risk score. Model also included main effects of exposome score and of depression polygenic risk score. The graph plots the probability of belonging to the depression-susceptible class along depression polygenic risk, moderated by exposomic burden (categorized as low, medium, or high), which demonstrates the interaction effect between major depressive disorder (MDD) polygenic risk score and the adverse environment (exposome score). Percentage probability of susceptibility denotes the probability of the youths belonging to the depression-susceptible class as opposed to the resilient class. The probability curve bands denote 95% CIs.

In European ancestry participants, we observed a significant gene-environment interaction (OR, 0.72; 95% CI, 0.56-0.93; *P* = .01) (Figure 4; eTable 17 in Supplement 1), whereby polygenic risk was associated with depression-susceptibility class membership only in youths with low exposomic risk, suggesting genetic vulnerability is primarily associated with risk in the absence of significant environmental adversity. The interactive association was not observed in African ancestry youths (OR, 1.01; 95% CI, 0.71-1.42; *P* = .97) (eTable 18 in Supplement 1).

### Sensitivity Analyses

Depression symptom trajectories were similar to main analyses when applying broader definitions of depressive symptoms (eFigure 3 in Supplement 1) and when using imputed depressive symptom data (eFigure 4 in Supplement 1). The depression-susceptible class slopes were significant in both broader-definition and imputed symptom score models, as in main analysis (eTables 19 and 20 in Supplement 1), suggesting that the emergence of trajectory classes did not depend on our symptom definitions or on data missingness. When accounting for variability in 2 pandemic stressors, financial strain and family conflict, all prepandemic risk and resilience factors remained associated with susceptibility with the same direction and statistical significance, apart from the change in significance of the associations with in-person peer bullying when adjusting for each stressor, late puberty when adjusting for family conflict, and polygenic risk when adjusting for family conflict (eFigures 5 and 6 in Supplement 1).

## Discussion

In this cohort study of a large and diverse community sample of US adolescents, we delineated variability in depressive symptom trajectories from before to after the COVID-19 pandemic and identified prospective prepandemic risk and resilience factors. In line with prepandemic literature on mental health after adversity,^15,33,35,76^ most (>85%) youths demonstrated a resilient low symptom trajectory, whereas a minority (<10%) showed increase in depression symptoms from before to after the pandemic. Female sex and late pubertal or postpubertal stage before the pandemic were associated with depression susceptibility, as were several multidimensional prepandemic risk (online and offline peer bullying, family conflict, maternal history, and polygenic risk of depression) and resilience (parental monitoring and problem-solving skills) factors.^2,10,34^ Results reaffirm the multilayered nature of risk and resilience across biopsychosocial dimensions that contribute to the course of mental health and explain heterogeneity in adolescent depression onset around an adverse event.^10^

Our findings can help classify youths at risk for depression after highly disruptive adverse events. The observed greater depression susceptibility among girls and youths in late puberty or postpuberty before the pandemic align with the reported increased prevalence of depression and anxiety in girls and older youths during the first stages of the pandemic.^2,3,77^ Epidemiological studies have highlighted that during the early phases of the pandemic, the prevalence of adolescent depression in the US increased from 4.15% in 2017 to 6.88% in 2021.^77^ Notably, meta-analyses of depression incidence have suggested incidence of emerging adolescent depression of approximately 20%^2,3^; however, they only included studies from early in the pandemic (up to 2021). We expand on these results by incorporating longitudinal data from before, during, and after the pandemic up to 2022 using a person-specific trajectory analysis approach, and by showing that pubertal stage is significantly associated with susceptibility to depression throughout and, consequently, after the pandemic. From a clinical standpoint, these findings suggest that among individuals who lived through the pandemic as adolescents, those at a later pubertal stage are at increased risk for depression and may thus benefit from additional screening and potential early intervention.^18,29^ The social, psychological, and biological dimensions of puberty have been extensively linked to depressive symptoms,^78,79,80,81^ and our findings reiterate their essential role in the emergence of depressive psychopathology over this developmental stage. Our findings regarding resilience factors may suggest that targeting modifiable resilience mechanisms, such as parental involvement and youth problem-solving skills, may reduce depression risk in teens going through a chronic stressor. Notably, preliminary trials demonstrate feasibility of reducing adolescents’ depressive symptoms through interventions that enhance parental involvement (including forms of monitoring).^82^ Likewise, randomized clinical trials of interventions promoting problem-solving skills early in the pandemic have shown positive effects on mental health.^83^

We found that several prepandemic individual environmental adversities across multiple levels were associated with depression-susceptible trajectory, including familial conflict, maternal history of depression, and peer aggression, consistent with the ecological systems theory that highlights the critical contribution of multilevel environmental exposures to human development.^10,45,84,85^ Our findings reaffirm the role of the environment in depression risk in adolescence, beyond sex, puberty, and genetic factors.^63,86,87^ Note that our analyses revealed no differences by race between resilience and depression-susceptible trajectories, supporting previous work in ABCD Study reporting that it is the exposome, rather than the racial identity itself, which contributes to mental health disparities.^88,89^ By incorporating polygenic risk score of depression and an aggregate polyenvironmental (exposomic) measure of prepandemic adversity, we were able to longitudinally explore the combined genetic and environmental contribution to depression trajectories around the pandemic. The observed gene-environment interaction, whereby genetic risk is associated with susceptibility only at low exposomic burden, suggests that genetic associations depend on prior adversity, aligning with the differential susceptibility hypothesis (ie, individuals vary in their responsiveness to experiences, for better or worse),^90,91,92,93^ and may underlie interindividual differences in the influence of polygenic risk on outcomes,^94,95^ particularly for depression.^96^ These findings strengthen empirical support for the interplay of genes, environment, and their interaction in adolescent depression.

### Limitations

This work should be interpreted considering certain limitations. First, our symptom score included only 6 of the 9 *DSM-5* depression symptoms. Nonetheless, the high rate of K-SADS–confirmed depression diagnoses (56.2%) within the depression-susceptible class supports our approach’s clinical validity; notably, K-SADS is a criterion-standard, semistructured interview with strong evidence for reliability and validity in diagnosing depression in youths.^38^ Second, because all youths in the cohort experienced the pandemic during adolescence, one cannot parse whether the observed depressive symptom trajectories are specifically related to pandemic-related stress or are part of typical adolescent development, a period of increased depression risk.^62,97^ We, therefore, are careful not to attribute the findings exclusively to the pandemic stress or assume equal pandemic-adversity exposure burden; instead, we use the pandemic as an example for adolescent development during chronic stress of variable intensity.^98^ Moreover, our findings should not be interpreted as directly demonstrating pandemic-specific associations, but rather variability in depression symptoms in a period spanning the pandemic. Relatedly, we did not consider differences in pandemic experience when modeling trajectories, which differed across geographic locations (eg, differences in policy regarding in-person vs remote school), socioeconomic classes, and minoritized groups, exacerbating preexisting inequalities.^99^ These factors contributed to variability in mental health in ABCD Study participants in earlier phases of the pandemic, as we and others have shown.^20,31,32^ Notably, our sensitivity analyses that adjusted for financial strain and family conflict during the pandemic were consistent with main analyses that did not account for pandemic stressors, suggesting that the prepandemic risk and resilience factors were not dependent on the individual pandemic experiences. More research is needed to address the role of the COVID-19 exposome, the scope of experiences throughout the pandemic (eg, change from in-person to virtual school and change in screen time), in determining mental health outcomes after the pandemic. Third, although we included polygenic risk models for diverse genetic ancestries, our analyses showed significant results only in European-ancestry youths. The reduced number of African-ancestry participants may have constrained statistical power, resulting in no significant associations. Fourth, our results are derived from a single cohort, restricting external validity and generalizability to broader adolescent populations. Future research should examine post–COVID-19 depression trajectories in other youth samples.

## Conclusions

In conclusion, this study supports prepandemic observations that most adolescents remain resilient to mental health burden following adversity. The results highlight multiple risk and resilience factors associated with interindividual variability in depressive symptom trajectories from before to after the COVID-19 pandemic. Findings may guide depression risk assessment among adolescents living through chronic disruptive stressors, highlighting parental monitoring and youth problem-solving skills as potential modifiable resilience factors and intervention targets. Further research is needed to examine long-term outcomes of individuals who experienced the pandemic during early adolescence as they transition into late adolescence and adulthood, and test how their unique pandemic experiences shape mental health trajectories.

## Notes

Supplement 1. eAppendix 1. Calculation of outcome measure: depression symptom score eAppendix 2. Pre-pandemic risk and resilience factors’ measurements eReferences eTable 1. High specificity, low sensitivity depression score definitions eTable 2. Low specificity, high sensitivity depression score definitions eTable 3. Missingness of depression score across study timepoints eTable 4. Latent growth mixed model fit statistics Depressive symptoms’ trajectories eTable 5. Intercept and slope statistics of the three-class trajectories model eTable 6. Comparisons of post-pandemic mental health diagnoses between resilient and depression-susceptible trajectories eTable 7. Demographic comparison of resilient, susceptible, and chronic trajectory groups eTable 8. Associations of late/post pubertal stage (binary measure) with depressive symptom trajectory class eTable 9. Associations of pubertal stage (5-point Likert scale) with depressive symptom trajectory class eTable 10. Depression trajectory models stratified by pubertal stage throughout the pandemic eTable 11. Associations of pre-pandemic prosocial behaviors and problem-solving skills with depressive symptom trajectory class eTable 12. Associations of family environment with depressive symptom trajectory class eTable 13. Associations of peer-victimization measures with depressive symptom trajectory class eTable 14. Associations of parental history of depression with depressive symptom trajectory class eTable 15. Associations of pre-pandemic poly-environmental (exposome) adversity score and socioeconomic measures with depressive symptom trajectory class eTable 16. Associations of polygenic risk of depression with depressive symptom trajectories among European-like or African-like genetic ancestry youth eTable 17. Main and interactive associations of exposomic risk and polygenic risk of depression with depressive symptom trajectories among European-like ancestry youth eTable 18. Main and interactive associations of exposomic risk and polygenic risk of depression with depressive symptom trajectory class among African-like ancestry youth eTable 19. Trajectory model’s intercept and slope statistics when using sensitive criteria of depressive symptoms eTable 20. Trajectory model’s intercept and slope statistics when using imputed symptom scores eFigure 1.Timeline of COVID-19 pandemic events during data collection period eFigure 2. Puberty-related differences between resilient and depression-susceptible trajectory classes eFigure 3. Visualization depressive symptom scores trajectories using sensitive criteria of depressive symptoms eFigure 4. Visualization depressive symptom scores trajectories using imputed symptom scores eFigure 5. Multi-level prospectively measured pre-pandemic risk and resilience factors, when adjusting for parent-reported family conflict during the pandemic eFigure 6. Multi-level prospectively measured pre-pandemic risk and resilience factors, when adjusting for parent-reported financial strain during the pandemic

Supplement 2. Data Sharing Statement
