# Supplementary material for: Adolescent Depressive Symptom Trajectories From Before to After the COVID-19 Pandemic
Source: JAMA Netw Open. 2025 Dec 1;8(12):e2545987. doi: 10.1001/jamanetworkopen.2025.45987 (PMC12670201; doi:10.1001/jamanetworkopen.2025.45987)
Supplement: Supplement 1. — eAppendix 1. Calculation of outcome measure: depression symptom score eAppendix 2. Pre-pandemic risk and resilience factors’ measurements eReferences eTable 1. High specificity, low sensitivity depression score definitions eTable 2. Low specificity, high sensitivity depression score definitions eTable 3. Missingness of depression score across study timepoints eTable 4. Latent growth mixed model fit statistics Depressive symptoms’ trajectories eTable 5. Intercept and slope statistics of the three-class trajectories model eTable 6. Comparisons of post-pandemic mental health diagnoses between resilient and depression-susceptible trajectories eTable 7. Demographic comparison of resilient, susceptible, and chronic trajectory groups eTable 8. Associations of late/post pubertal stage (binary measure) with depressive symptom trajectory class eTable 9. Associations of pubertal stage (5-point Likert scale) with depressive symptom trajectory class eTable 10. Depression trajectory models stratified by pubertal stage throughout the pandemic eTable 11. Associations of pre-pandemic prosocial behaviors and problem-solving skills with depressive symptom trajectory class eTable 12. Associations of family environment with depressive symptom trajectory class eTable 13. Associations of peer-victimization measures with depressive symptom trajectory class eTable 14. Associations of parental history of depression with depressive symptom trajectory class eTable 15. Associations of pre-pandemic poly-environmental (exposome) adversity score and socioeconomic measures with depressive symptom trajectory class eTable 16. Associations of polygenic risk of depression with depressive symptom trajectories among European-like or African-like genetic ancestry youth eTable 17. Main and interactive associations of exposomic risk and polygenic risk of depression with depressive symptom trajectories among European-like ancestry youth eTable 18. Main and interactive associations of exposomic risk and polyg [file jamanetwopen-e2545987-s001.pdf]

Supplemental Online Content

Gataviņš MM, Tran TT, Visoki E, et al. Adolescent depressive symptom trajectories from before to after the COVID-19 pandemic. *JAMA Netw Open*. 2025;8(12):e2545987. doi:10.1001/jamanetworkopen.2025.45987

eAppendix 1. Calculation of outcome measure: depression symptom score
eAppendix 2. Pre-pandemic risk and resilience factors' measurements
eReferences
eTable 1. High specificity, low sensitivity depression score definitions
eTable 2. Low specificity, high sensitivity depression score definitions
eTable 3. Missingness of depression score across study timepoints
eTable 4. Latent growth mixed model fit statistics depressive symptoms' trajectories
eTable 5. Intercept and slope statistics of the three-class trajectories model
eTable 6. Comparisons of post-pandemic mental health diagnoses between resilient and depression-susceptible trajectories
eTable 7. Demographic comparison of resilient, susceptible, and chronic trajectory groups
eTable 8. Associations of late/post pubertal stage (binary measure) with depressive symptom trajectory class
eTable 9. Associations of pubertal stage (5-point Likert scale) with depressive symptom trajectory class
eTable 10. Depression trajectory models stratified by pubertal stage throughout the pandemic
eTable 11. Associations of pre-pandemic prosocial behaviors and problem-solving skills with depressive symptom trajectory class
eTable 12. Associations of family environment with depressive symptom trajectory class
eTable 13. Associations of peer-victimization measures with depressive symptom trajectory class
eTable 14. Associations of parental history of depression with depressive symptom trajectory class
eTable 15. Associations of pre-pandemic poly-environmental (exposome) adversity score and socioeconomic measures with depressive symptom trajectory class
eTable 16. Associations of polygenic risk of depression with depressive symptom trajectories among European-like or African-like genetic ancestry youth
eTable 17. Main and interactive associations of exposomic risk and polygenic risk of depression with depressive symptom trajectories among European-like ancestry youth
eTable 18. Main and interactive associations of exposomic risk and polygenic risk of depression with depressive symptom trajectory class among African-like ancestry youth
eTable 19. Trajectory model's intercept and slope statistics when using sensitive criteria of depressive symptoms
eTable 20. Trajectory model's intercept and slope statistics when using imputed symptom scores
eFigure 1. Timeline of COVID-19 pandemic events during data collection period
eFigure 2. Puberty-related differences between resilient and depression-susceptible trajectory classes
eFigure 3. Visualization depressive symptom scores trajectories using sensitive criteria of depressive symptoms

© 2025 Gataviņš MM et al. *JAMA Network Open*.

eFigure 4. Visualization depressive symptom scores trajectories using imputed symptom scores
eFigure 5. Multi-level prospectively measured pre-pandemic risk and resilience factors, when adjusting for parent-reported family conflict during the pandemic
eFigure 6. Multi-level prospectively measured pre-pandemic risk and resilience factors, when adjusting for parent-reported financial strain during the pandemic

This supplemental material has been provided by the authors to give readers additional information about their work.

© 2025 Gataviņš MM et al. *JAMA Network Open*.

eAppendix 1. Calculation of outcome measure: depression symptom score

Given the difference in the mental health instruments administered in the ABCD Study® follow-ups (ABCD main study) and COVID-19 Rapid Research Response (RRR) survey (ABCD COVID-19 sub-study; CV) assessments, we created a symptom-specific depression score that combines different measures from instruments in the main and COVID-19 sub-study assessments at each timepoint to derive a relative broad-spectrum depression score. Across the main and COVID-19 sub-study assessment instruments, we identified six out of nine DSM-V (Diagnostic and Statistical Manual of Mental Disorders, 5th Edition) Major Depressive Disorder (termed hereafter depression) symptoms to be represented: depressive mood (A1), anhedonia (A2), sleep problems (A4), fatigue and lack of energy (A6), sense of guilt and worthlessness (A7), and attention and concentration problems (A8). By consensus among three child and adolescent psychiatrists (KWH, TS, RB), questions from each scale were categorized into one of six DSM symptoms. Thresholds were established by agreement among all three clinicians or at least two of them to determine the presence of a symptom. This process resulted in two coding schemes: one with high specificity and low sensitivity ("specific") and another with low specificity and high sensitivity ("sensitive") scores

From the main study, we used questions from the Brief Problem Monitor for Youth (BPM-Y),[1] the Munich Chronotype Questionnaire (MCTQ),[2,3] the symptom scores derived from the computerized self-report Kiddie Schedule for Affective Disorders and Schizophrenia (K-SADS) Screening[4,5] and Depression modules,[6] and NIH Emotion Toolbox Positive Affect Scale.[7,8] From the COVID-19 RRR Survey data, we used questions from the NIH Emotion Toolbox Positive and Sadness Scales[7–10] and the

© 2025 Gataviņš MM et al. *JAMA Network Open*.

shortened 4-question MCTQ.[2,10] Each question was binarized according to a criteria set. Binarization criteria are available in **Supplemental Tables S1** and **S2** for high specificity low sensitivity ("specific"), and low specificity high sensitivity ("sensitive") scores, respectively.

A depressive symptom counted as 'endorsed' (1) if any symptom criteria were met. If none of the questions counted as 'endorsed' and all questions from the administered instruments at the timepoint were not missing, the depressive symptom counted as 'not endorsed' (0). For questions with conditional administration (i.e., K-SADS depression module questions, which are administered only if the participant meets K-SADS Screening criteria for further assessment), if the questions were not administered by condition, they were counted as 'not endorsed' (0). Not all six symptoms were available in all bins. For example, for some participants, the pre-pandemic data did not include a K-SADS (those with 1-year follow-up assessment as pre-pandemic data) or BPM assessment (those with baseline assessment as pre-pandemic data); for some participants, the post pandemic data was derived from the 3-year follow-up assessment, which did not include K-SADS.

Kiddie Schedule for Affective Disorders & Schizophrenia

ABCD Study administers a standardized, computerized self-report Kiddie Schedule for Affective Disorders & Schizophrenia.[4] The questions are asked using a branching logic, whereby an initial Screening module is used to determine what further psychopathology dimension-specific modules are to be administered. In our study, we used questions from the Screening and Depression module. The Depression module is

administered if threshold score is meet for any one of the depression screening question categories:[6]

- Depressed mood: felt down, depressed (e.g., Have you ever felt sad, blue, down, or empty?)
- Irritability and anger: felt more irritated than usual (e.g., Did you ever have a time where you lost your temper a lot? When was that?)
- Anhedonia, lack of interest, apathy, low motivation, or boredom: has less fun doing things (e.g., Did you feel bored while you were doing things you used to enjoy?)
- Suicidality questions

Brief Problem Monitor

Brief Problem Monitor is a short-form, self-report instrument covering general psychopathology and is based on the long-form Child Behavior Checklist.[1] BPM covers internalizing, externalizing, and attention psychopathology.

NIH Emotion Toolbox: Positive and Negative Affect Scale

NIH Emotion Toolbox is a battery of instruments covering affect and emotional disposition in the week preceding the time of administration.[7] In the main study, only a short-form Positive Affect Scale is administered, while for the COVID-19 RRR survey Positive Affect, Sadness, and Fear scales are administered.[9,11,12] Previous studies using COVID-19 RRR survey data have used measures from the Positive Affect and Sadness Scales to operationalize dimensions of depressive symptomatology and have shown

© 2025 Gataviņš MM et al. *JAMA Network Open*.

consistent associations with COVID-19 stressors, specifically, financial strain and social determinants of health.[10–13]

Munich Chronotype Questionnaire

Munich Chronotype Questionnaire is an instrument assessing sleep quality, specifically amount, inertia, and latency.[2,3] In the main study, MCTQ is assessed both during school days and days off school; however, the COVID-19 RRR survey MCTQ is assessed independent of school days.[10] Because of this, for the main study MCTQ, we calculated a mean to create general measures of MCTQ, which have been previously used in studies of COVID-19 sleep quality and have been associated with COVID-19 specific stressors and social determinants of health.[10]

Calculating time-period bins

To account for the heterogeneity in the administration of the assessments throughout COVID timeframe (see **Main Figure 1** for collection date distribution), we created six time bins, delineating the average depressive symptom score within that time period. All pre-March 2020 and all post-July 2021 main study assessments were categorized into pre- and post-pandemic bins respectively. We created four separate bins throughout March 2020 and July 2021: (1) March – July 2020, (2) August – November 2020, (3) December 2020 – April 2021, and (4) May – July 2021.

Depressive symptom scores were calculated at each assessment separate from other assessments (i.e., irrespective of the date of administration). For the pre- and post-COVID time bins we used the latest pre-COVID and earliest post-COVID time

© 2025 Gataviņš MM et al. *JAMA Network Open*.

assessments; for pandemic bins (between March 2020 and July 2021), we averaged all scores for assessments that were administered within time period of the respective bin.

Handling missingness due to administration of instruments

In the COVID-19 RRR Survey, NIH Sadness Scale and Positive Affect Scales were administered at different assessment timepoints – the former during surveys 1, 3, 5, and 7, the latter during surveys 2, 4, and 6. Given the COVID 19 sub-study surveys were administer at a higher time resolution (as opposed to the yearly follow-ups for main study), we addressed this by using either the adjacent timepoint or the mean of two adjacent scores. For example, for the Positive Affect Scale measures, timepoints 1 and 7 were replaced with the measures from timepoints 2 and 6, respectively. For timepoint 3, we used the mean of timepoints 2 and 4, and for timepoint 5, we used the mean of timepoints 4 and 6.

Imputation to account for missingness

The imputation process in this analysis addresses missing data in the items used to calculate depression symptom scores across multiple assessments (baseline, 1-year, 2-year, 3-year, and 4-year follow-ups) by applying the missForest package in R. The dataset was split by timepoint, and imputation is performed separately for each timepoint. In the main study, individual item data were imputed only for those items that were administered at that follow-up timepoint. However, for the COVID-19 depression symptoms, imputation was not performed separately by timepoint. Instead, missing data

across all timepoints for the COVID-19 RRR depression symptoms were imputed jointly, using the same missForest package to handle missing values across the entire dataset.

© 2025 Gataviņš MM et al. *JAMA Network Open*.

eAppendix 2. Pre-pandemic risk and resilience factors' measurements

To encapsulate the multiple dimensions of resilience, as postulated by ecological theories of development and resilience, we used a broad scope of measures that differed by dimension of environment and resilience, administration, and level of measurement. In total, we included 13 prospectively measured variables: pubertal stage, problem-solving skills, prosocial behaviors, parental history of depression, family conflict, parental monitoring, cyberbullying, peer victimization, household income, Area Deprivation Index (ADI), exposome score,[14] and two different polygenic risk scores for depression (for European-like[15] and African-like[16] genetic ancestry).

Pubertal stage: Pubertal Development Scale

The Pubertal Development Scale (PDS) was administered to both participants and their guardians and assessed physical changes associated with puberty: height spurts, skin changes, body hair, breast development and menstruation for girls and voice deepening and facial hair growth for boys.[17] Based on the sum of three 4-point Likert scale questions (from no observed changes [1] to complete growth [4]), participants were then designated an approximate pubertal stage (pre- [1], early, mid-, late, or post-pubertal [5]). We used the child self-report form. The PDS was administered annually since baseline assessment. Throughout analyses, we binarized puberty into early and late/post-puberty, whereby the former included PDS scores below 3 and the latter PDS of 4 and 5. Additional sensitivity analyses were performed using all five stages separately as opposed to binarized puberty.

© 2025 Gataviņš MM et al. *JAMA Network Open*.

Problem-solving skills: Wills' Problem-Solving Scale

Problem-solving skills were quantified using the Wills Problem Solving Scale (WPSS), a six-item self-report questionnaire answered on a five-point response scale (Never = 1 to Usually = 5).[18,19] WPSS was administered at the one and three-year follow-ups.

Prosocial behaviors: Prosocial Behavior Scale

We quantified prosocial behaviors using the average score of the youth-administered Prosocial Behavior Scale (PBS) as part of the Strengths and Difficulties Questionnaire.[20–22] The ABCD version of the instrument has three items rated on a three-point response scale (0 = Not True to 2 = Certainly True). The PBS was administered annually since baseline assessment.

Family history of psychopathology

To assess the effects of familial depression history, we used parent-reported information on mental health history, specifically using the responses on biological parent history of depression.

Family environment: conflict and parental monitoring

Family conflict was quantified using the ABCD Youth Family Environment Scale – Conflict Subscale (a sum score).[23–25] The subscale consists of 9 yes/no questions (endorsing whether a certain behavior is observed in the family).

© 2025 Gataviņš MM et al. *JAMA Network Open*.

Youth-reported parental monitoring was measured using the five-item ABCD Parental Monitoring scale, with each item rated on a 5-point Likert scale (1 = Never to 5 = Always or Almost Always).[26,27]

Both scales were administered annually, i.e., at all pre-COVID timepoints (baseline to three-year).

Peer victimization: cyberbullying and peer victimization

To capture peer relationships, specifically their negative effects, we quantified peer victimization, both cyberbullying and offline experiences of peer victimization, using measures previously used by our research group to characterize relationships between bullying and suicidality.[28]

The cyberbullying measure was taken from the ABCD Cyber Bullying Questionnaire. We used a binary response to the question “Have you ever been cyberbullied, where someone was trying on purpose to harm you or be mean to you online, in texts, or group texts, or on social media (like Instagram or Snapchat)?”[29]

The extent of offline experiences of peer victimization was defined using the Peer Experiences Questionnaire,[30] which captures three dimensions of peer violence, with three questions for each dimension, and their frequency using a five-point Likert scale from 1 (“never”) to 5 (“a few times a week”). Each dimension was scored between 3 and 15 and consistent with previous work, we calculated a sum score of all three dimensions and dichotomized the measure by top decile, thereby creating a binary variable that defined youth that experience high peer victimization (were in the top decile).[28] Both questionnaires were administered annually since 2-year follow-up.

© 2025 Gataviņš MM et al. *JAMA Network Open*.

Household income

In the ABCD Study, household income is quantified into ten bins of annual household income, which we treated as a continuous variable: 1= Less than $5,000; 2=$5,000 through $11,999; 3=$12,000 through $15,999; 4=$16,000 through $24,999; 5=$25,000 through $34,999; 6=$35,000 through $49,999; 7=$50,000 through $74,999; 8= $75,000 through $99,999; 9=$100,000 through $199,999; 10=$200,000 and greater.

Area Deprivation Index

We chose the Area Deprivation Index (ADI) to quantify structural inequality and socioeconomic status, which is location-linked to the census block group of the participant's address. ADI is a measure created by the Health Resources & Service Administration and uses American Community Survey 5-year data to quantify income, education, employment, and housing quality.[31,32] The score is a measure of relative socioeconomic disadvantage across the United States, whereby a higher score denotes higher disadvantage (deprivation).

Exposome score

We used a general adversity exposome score that we have previously calculated through dimensionality reduction of 348 environmental variables collected by the one-year follow-up. Further information on method of calculation and validation is available in our previous literature.[14]

© 2025 Gataviņš MM et al. *JAMA Network Open*.

Briefly, we conducted iterative exploratory factor analyses on multi-level environmental measures in six domains (household adversity, neighborhood environment, day-to-day experiences, state environment, family values and pregnancy/birth complications). Thereafter, we estimated a bifactor model, generating a single exposome factor score (*z*-score) that captures the shared variance of the adverse environment.

Polygenic risk scores

We calculated polygenic risk scores (PRSs) for depression using summary statistics from depression genome-wide-association-studies (GWAS),[15,16] as previously described in prior works.[33,34] PRSs were calculated for participants with genetic similarity to European ancestry (EUR) and African ancestry (AFR) using the respective EUR[15] and AFR GWAS.[16]

Briefly, PRS-CS (PRS using single-nucleotide polymorphism (SNP) effect sizes under continuous shrinkage)[35] was used to infer the posterior effect sizes of SNPs in the dataset that overlapped with the GWAS summary statistics, and an external 1000 Genomes linkage disequilibrium (LD) panel matched to the genetic similarity reference group used for the GWAS. Raw PRSs were produced by PLINK v.1.9 and then standardized in R. The first ten genetic principal components were regressed out of the standardized PRS.

© 2025 Gataviņš MM et al. *JAMA Network Open*.

eReferences

1. Achenbach TM, McConaughy SH, Ivanova MY, Rescorla LA. Manual for the ASEBA brief problem monitor (BPM). *Burlingt VT ASEBA*. 2011;33.

2. Roenneberg T, Pilz LK, Zerbini G, Winnebeck EC. Chronotype and Social Jetlag: A (Self-) Critical Review. *Biology*. 2019;8(3). doi:10.3390/BIOLOGY8030054

3. Roenneberg T, Wirz-Justice A, Merrow M. Life between clocks: daily temporal patterns of human chronotypes. *J Biol Rhythms*. 2003;18(1):80-90. doi:10.1177/0748730402239679

4. Townsend L, Kobak K, Kearney C, et al. Development of Three Web-Based Computerized Versions of the Kiddie Schedule for Affective Disorders and Schizophrenia Child Psychiatric Diagnostic Interview: Preliminary Validity Data. *J Am Acad Child Adolesc Psychiatry*. 2020;59(2):309-325. doi:10.1016/j.jaac.2019.05.009

5. Kaufman J, Birmaher B, Brent D, et al. Schedule for Affective Disorders and Schizophrenia for School-Age Children-Present and Lifetime Version (K-SADS-PL): Initial Reliability and Validity Data. *J Am Acad Child Adolesc Psychiatry*. 1997;36(7):980-988. doi:10.1097/00004583-199707000-00021

6. Hamilton J, Gillham J. THE K-SADS AND DIAGNOSIS OF MAJOR DEPRESSIVE DISORDER. *J Am Acad Child Adolesc Psychiatry*. 1999;38(9):1065-1066. doi:10.1097/00004583-199909000-00001

7. Salsman JM, Butt Z, Pilkonis PA, et al. Emotion assessment using the NIH Toolbox. *Neurology*. 2013;80(11 Suppl 3):S76-S76. doi:10.1212/WNL.0B013E3182872E11

8. Karcher NR, Barch DM. The ABCD study: understanding the development of risk for mental and physical health outcomes. *Neuropsychopharmacol 2020 461*. 2020;46(1):131-142. doi:10.1038/s41386-020-0736-6

9. Zhang L, Cropley VL, Whittle S, Rakesh D. Adolescent resilience in the face of COVID-19 stressors: the role of trauma and protective factors. *Psychol Med*. Published online October 14, 2024:1-11. doi:10.1017/S0033291724001806

10. Xiao Y, Brown TT, Snowden LR, Chow JCC, Mann JJ. COVID-19 Policies, Pandemic Disruptions, and Changes in Child Mental Health and Sleep in the United States. *JAMA Netw Open*. 2023;6(3):e232716-e232716. doi:10.1001/JAMANETWORKOPEN.2023.2716

11. Argabright ST, Tran KT, Visoki E, DiDomenico GE, Moore TM, Barzilay R. COVID-19-related financial strain and adolescent mental health. *Lancet Reg Health - Am*. 2022;16. doi:10.1016/j.lana.2022.100391

12. Stinson EA, Sullivan RM, Peteet BJ, et al. Longitudinal Impact of Childhood Adversity on Early Adolescent Mental Health During the COVID-19 Pandemic in the ABCD Study Cohort: Does Race or Ethnicity Moderate Findings? *Biol Psychiatry Glob Open Sci*. 2021;1(4):324-324. doi:10.1016/J.BPSGOS.2021.08.007

© 2025 Gataviņš MM et al. *JAMA Network Open*.

13. Xiao Y, Yip PSF, Pathak J, Mann JJ. Association of Social Determinants of Health and Vaccinations With Child Mental Health During the COVID-19 Pandemic in the US. *JAMA Psychiatry*. 2022;79(6):610-621. doi:10.1001/JAMAPSYCHIATRY.2022.0818

14. Moore TM, Visoki E, Argabright ST, et al. Modeling environment through a general exposome factor in two independent adolescent cohorts. *Exposome*. 2022;2(1):osac010. doi:10.1093/exposome/osac010

15. Howard DM, Adams MJ, Clarke TK, et al. Genome-wide meta-analysis of depression identifies 102 independent variants and highlights the importance of the prefrontal brain regions. *Nat Neurosci*. 2019;22(3):343-352. doi:10.1038/s41593-018-0326-7

16. Levey DF, Stein MB, Wendt FR, et al. Bi-ancestral depression GWAS in the Million Veteran Program and meta-analysis in >1.2 million individuals highlight new therapeutic directions. *Nat Neurosci*. 2021;24(7):954-963. doi:10.1038/s41593-021-00860-2

17. Cheng TW, Magis-Weinberg L, Guazzelli Williamson V, et al. A Researcher's Guide to the Measurement and Modeling of Puberty in the ABCD Study® at Baseline. *Front Endocrinol*. 2021;12(May). doi:10.3389/fendo.2021.608575

18. Wills TA, Dishion TJ. Temperament and Adolescent Substance Use: A Transactional Analysis of Emerging Self-Control. *J Clin Child Adolesc Psychol*. 2004;33(1):69-81. doi:10.1207/S15374424JCCP3301_7

19. Wills TA, Windle M, Cleary SD. Temperament and novelty seeking in adolescent substance use: Convergence of dimensions of temperament with constructs from Cloninger's theory. *J Pers Soc Psychol*. 1998;74(2):387-406. doi:10.1037/0022-3514.74.2.387

20. Goodman R, Meltzer H, Bailey V. The Strengths and Difficulties Questionnaire: A pilot study on the validity of the self-report version. *Eur Child Adolesc Psychiatry*. 1998;7(3):125-130.

21. Goodman R, Scott S. Comparing the Strengths and Difficulties Questionnaire and the Child Behavior Checklist: is small beautiful? *J Abnorm Child Psychol*. 1999;27:17-24.

22. Barch DM, Albaugh MD, Baskin-Sommers A, et al. Demographic and mental health assessments in the adolescent brain and cognitive development study: Updates and age-related trajectories. *Dev Cogn Neurosci*. 2021;52. doi:10.1016/J.DCN.2021.101031

23. Gonzalez R, Thompson EL, Sanchez M, et al. An update on the assessment of culture and environment in the ABCD Study®: Emerging literature and protocol updates over three measurement waves. *Dev Cogn Neurosci*. 2021;52:101021-101021. doi:10.1016/J.DCN.2021.101021

24. Moos RH, Moos BS. A Typology of Family Social Environments. *Fam Process*. 1976;15(4):357-371. doi:10.1111/j.1545-5300.1976.00357.x

© 2025 Gataviņš MM et al. *JAMA Network Open*.

25. Zucker RA, Gonzalez R, Feldstein Ewing SW, et al. Assessment of culture and environment in the Adolescent Brain and Cognitive Development Study: Rationale, description of measures, and early data. *Dev Cogn Neurosci*. 2018;32:107-120. doi:10.1016/j.dcn.2018.03.004

26. Keller AS, Mackey AP, Pines A, et al. Caregiver monitoring, but not caregiver warmth, is associated with general cognition in two large sub-samples of youth. *Dev Sci*. 2023;26(3):e13337. doi:10.1111/desc.13337

27. Karoly HC, Callahan T, Schmiege SJ, Feldstein Ewing SW. Evaluating the Hispanic Paradox in the Context of Adolescent Risky Sexual Behavior: The Role of Parent Monitoring. *J Pediatr Psychol*. 2016;41(4):429-440. doi:10.1093/jpepsy/jsv039

28. Arnon S, Brunstein Klomek A, Visoki E, et al. Association of Cyberbullying Experiences and Perpetration With Suicidality in Early Adolescence. *JAMA Netw Open*. 2022;5(6):e2218746. doi:10.1001/jamanetworkopen.2022.18746

29. Hoffman EA, Clark DB, Orendain N, Hudziak J, Squeglia LM, Dowling GJ. Stress exposures, neurodevelopment and health measures in the ABCD study. *Neurobiol Stress*. 2019;10:100157. doi:10.1016/j.ynstr.2019.100157

30. Prinstein MJ, Boergers J, Vernberg EM. Overt and Relational Aggression in Adolescents: Social-Psychological Adjustment of Aggressors and Victims. *J Clin Child Adolesc Psychol*. 2001;30(4):479-491. doi:10.1207/S15374424JCCP3004_05

31. Kind AJH, Buckingham WR. Making Neighborhood-Disadvantage Metrics Accessible — The Neighborhood Atlas. *N Engl J Med*. 2018;378(26):2456-2458. doi:10.1056/NEJMp1802313

32. The Area Deprivation Index Is The Most Scientifically Validated Social Exposome Tool Available For Policies Advancing Health Equity. Published online July 20, 2023. doi:10.1377/forefront.20230714.676093

33. Daskalakis NP, Schultz LM, Visoki E, et al. Contributions of PTSD polygenic risk and environmental stress to suicidality in preadolescents. *Neurobiol Stress*. 2021;15:100411-100411. doi:10.1016/j.ynstr.2021.100411

34. Schultz LM, Merikangas AK, Ruparel K, et al. Stability of polygenic scores across discovery genome-wide association studies. *Hum Genet Genomics Adv*. 2022;3(2):100091. doi:10.1016/j.xhgg.2022.100091

35. Ge T, Chen CY, Ni Y, Feng YCA, Smoller JW. Polygenic prediction via Bayesian regression and continuous shrinkage priors. *Nat Commun*. 2019;10(1):1776-1776. doi:10.1038/s41467-019-09718-5

eTable 1. High specificity, low sensitivity depression score definitions

Symptom	Instrument	Item ID	Item	Answer options	Endorsement criteria
**ABCD Main Study**					
A1: Depressive Mood	K-SADS Screening	ksads_1_1_t & ksads2_1_1_t	Symptom - Depressed Mood, Present		Endorsed (1)
		ksads_1_2_t & ksads2_1_2_t	Symptom - Depressed Mood, Past		Endorsed (1)
		ksads_1_3_t & ksads2_1_3_t	Symptom - Irritability, Present		Endorsed (1)
		ksads_1_4_t & ksads2_1_4_t	Symptom - Irritability, Past		Endorsed (1)
	BPM	bpm_18_y	I am unhappy, sad, or depressed	0 = Not True; 1 = Somewhat True; 2 = Very True	Very true (2)
A2: Anhe	K-SAD	ksads_1_5_t & ksads2_1_5_t	Symptom - Anhedonia, Present		Endorsed (1)

© 2025 Gataviņš MM et al. *JAMA Network Open*.

		ksads_1_6_t & ksads2_1_6_t	Symptom - Anhedonia, Past		Endorsed (1)
	K-SADS Depression	ksads_1_179_t & ksads2_1_170_t	Symptom - Hopeless, Present		Endorsed (1)
		ksads_1_180_t & ksads2_1_171_t	Symptom - Hopeless, Past		Endorsed (1)
A4: Sleep problems	MCTQ	mctq_sd_min_to_sleep_calc & mctq_fd_min_to_sleep_calc	Sleep latency, average of workday and freeday		1 hour or above
		mctq_sd_num_wake_up & mctq_fd_num_wake_up	Report on your typical sleep behavior over the past week.: After falling asleep, I wake up ____ times during the night.	0 = 0 ; 1 = 1 ; 2 = 2 ; 3 = 3 ; 4 = 4 ; 5 = 5 ; 6 = 6 ; 7 = 7 ; 8 = 8 ; 9 = 9 ; 10 = 10	3 or more times
A6: Fatig	K-SAD	ksads_1_159_t & ksads2_1_150_t	Symptom - Fatigue, Present		Endorsed (1)

© 2025 Gataviņš MM et al. *JAMA Network Open*.

		ksads_1_160_t & ksads2_1_151_t	Symptom - Fatigue, Past		Endorsed (1)
A7: Sense of guilt & worthlessness	BPM	bpm_9_y	I feel worthless or inferior Definition of inferior: less good	0 = Not True; 1 = Somewhat True; 2 = Very True	Very true (2)
	K-SADS Depression	ksads_1_177_t & ksads2_1_168_t	Symptom - Guilt, Present		Endorsed (1)
		ksads_1_178_t & ksads2_1_169_t	Symptom - Guilt, Past		Endorsed (1)
A8: Attention and concentration problems	BPM	bpm_4_y	I have trouble concentrating or paying attention	0 = Not True; 1 = Somewhat True; 2 = Very True	Very true (2)
**COVID-19 RRR Survey**					

© 2025 Gataviņš MM et al. *JAMA Network Open*.

A1: Depressive Mood	NIH Sadness Scale	felt_sad_cv	In the past week: I felt sad	1= Never; 2= Almost Never; 3= Sometimes; 4= Often; 5= Almost Always	Often (4) or above
		felt_always_sad	In the past week.: I could not stop feeling sad	1= Never; 2= Almost Never; 3= Sometimes; 4= Often; 5= Almost Always	Often (4) or above
		felt_unhappy_cv	In the past week.: I felt unhappy	1= Never; 2= Almost Never; 3= Sometimes; 4= Often; 5= Almost Always	Almost always (5)

© 2025 Gataviņš MM et al. *JAMA Network Open*.

A2: Anhedonia		felt_no_fun_cv	It was hard for me to have fun	1= Never; 2= Almost Never; 3= Sometimes; 4= Often; 5= Almost Always	Often (4) or above
A4: Sleep problems	MCTQ	mctq_fd_min_to_sleep_cv	Report on your typical sleep behavior over the past week.: I need _________ minutes to fall asleep.	0 = 0 ; 1 = 1 ; 2 = 2 ; 3 = 3 ; 4 = 4 ; 5 = 5 ; 6 = 6 ; 7 = 7 ; 8 = 8 ; 9 = 9 ; 10 = 10 ; 11 = 15 ; 12 = 20 ; 13 = 25 ; 14 = 30 ; 15 = 40 ; 16 = 50 ; 17 = 1 hour ; 18 = 1 hour 15 minute ; 19 = 1 hour 30 minute ; 20 = 1 hour 45 minute ; 21 = 2 hours ; 22 = 3 hours ; 23 = 4 hours	1 hour (17) or above
		mctq_fd_num_wake_up_cv	Report on your typical sleep behavior over the past week.: After falling asleep, I wake up ____ times during the night.	0 = 0 ; 1 = 1 ; 2 = 2 ; 3 = 3 ; 4 = 4 ; 5 = 5 ; 6 = 6 ; 7 = 7 ; 8 = 8 ; 9 = 9 ; 10 = 10	3 or more times

© 2025 Gataviņš MM et al. *JAMA Network Open*.

A6: Fatigue	NIH Positive Affect	energetic_y_cv	Please rate how each item describes you now or within the past week. I felt energetic	1 = Not true; 3 = Somewhat true; 5 = Very true	Not true (1)
A7: Sense of guilt & worthlessness	NIH Sadness Scale	felt_cv	In the past week.: I felt like I couldn't do anything right	1= Never; 2= Almost Never; 3= Sometimes; 4= Often; 5= Almost Always	Often (4) or above
A8: Attention and concentration problems	NIH Positive Affect	attentive_y_cv	Please rate how each item describes you now or within the past week: I felt attentive (that is, alert or able to pay attention)	1 = Not true; 3 = Somewhat true; 5 = Very true	Not true (1)

© 2025 Gataviņš MM et al. *JAMA Network Open*.

		concentrate_y_cv	Please rate how each item describes you now or within the past week: I felt able to concentrate	1 = Not true; 3 = Somewhat true; 5 = Very true	Not true (1)

© 2025 Gataviņš MM et al. *JAMA Network Open*.

eTable 2. Low specificity, high sensitivity depression score definitions

Symptom	Instrument		Item ID	Item	Answer options	Endorsement criteria
**ABCD Main Study**						
A1: Depressive Mood	K-SADS	Screening	ksads_1_1_t & ksads2_1_1_t	Symptom - Depressed Mood, Present		Endorsed (1)
			ksads_1_2_t & ksads2_1_2_t	Symptom - Depressed Mood, Past		Endorsed (1)
			ksads_1_3_t & ksads2_1_3_t	Symptom - Irritability, Present		Endorsed (1)
			ksads_1_4_t & ksads2_1_4_t	Symptom - Irritability, Past		Endorsed (1)
	BPM		bpm_18_y	I am unhappy, sad, or depressed	0 = Not True; 1 = Somewhat True; 2 = Very True	True (1) or very true (2)
A2: Anhedonia	K-SADS	Screeni	ksads_1_5_t & ksads2_1_5_t	Symptom - Anhedonia, Present		Endorsed (1)
			ksads_1_6_t & ksads2_1_6_t	Symptom - Anhedonia, Past		Endorsed (1)
	K-SADS	Depressio	ksads_1_179_t & ksads2_1_170_t	Symptom - Hopeless, Present		Endorsed (1)
			ksads_1_180_t & ksads2_1_171_t	Symptom - Hopeless, Past		Endorsed (1)

© 2025 Gataviņš MM et al. *JAMA Network Open*.

	NIH Positive Affect	poa_nihtb_6_y	I felt interested.	1 = Not True; 2 = Somewhat True; 3 = Very True	Not true (1)
A4: Sleep problems	MCTQ	mctq_sd_min_to_sleep_calc & mctq_fd_min_to_sleep_calc	Sleep latency, average of workday and freeday		1 hour or above

© 2025 Gatavinš MM et al. *JAMA Network Open*.

		mctq_sd_num_wake_up & mctq_fd_num_wake_up	Report on your typical sleep behavior over the past week.: After falling asleep, I wake up ____ times during the night.	0 = 0 ; 1 = 1 ; 2 = 2 ; 3 = 3 ; 4 = 4 ; 5 = 5 ; 6 = 6 ; 7 = 7 ; 8 = 8 ; 9 = 9 ; 10 = 10	2 or more times
A6: Fatigue	K-SADS Depression	ksads_1_159_t & ksads2_1_150_t	Symptom - Fatigue, Present		Endorsed (1)
		ksads_1_160_t & ksads2_1_151_t	Symptom - Fatigue, Past		Endorsed (1)
	NIH Positive	poa_nihtb_8_y	I felt energetic	1 = Not True; 2 = Somewhat True; 3 = Very True	Not true (1) or somewhat true (2)
A7: Sense of guilt & [illegible]	BPM	bpm_9_y	I feel worthless or inferior Definition of inferior: less good	0 = Not True; 1 = Somewhat True; 2 = Very True	Very true (2)
		bpm_12_y	I feel too guilty	0 = Not True; 1 = Somewhat True; 2 = Very True	Very true (2)
	K-SA	ksads_1_177_t & ksads2_1_168_t	Symptom - Guilt, Present		Endorsed (1)

© 2025 Gataviņš MM et al. *JAMA Network Open*.

		ksads_1_178_t & ksads2_1_169_t	Symptom - Guilt, Past		Endorsed (1)
		ksads_1_181_t & ksads2_1_172_t	Symptom - Decreased Self-Esteem, Present		Endorsed (1)
		ksads_1_182_t & ksads2_1_173_t	Symptom - Decreased Self-Esteem, Past		Endorsed (1)
A8: Attention and concentration problems	BPM	bpm_4_y	I have trouble concentrating or paying attention	0 = Not True; 1 = Somewhat True; 2 = Very True	Very true (2)
	KSADS Depression	ksads_1_163_t & ksads2_1_154_t	Symptom - Indecision, Present		Endorsed (1)
		ksads_1_164_t & ksads2_1_155_t	Symptom - Indecision, Past		Endorsed (1)
	NIH Positive	poa_nihtb_1_y	I felt attentive (that is, alert or able to pay attention).	1 = Not True; 2 = Somewhat True; 3 = Very True	Not true (1) or somewhat true (2)

© 2025 Gataviņš MM et al. *JAMA Network Open*.

		poa_nihtb_9_y	I felt able to concentrate.	1 = Not True; 2 = Somewhat True; 3 = Very True	Not true (1) or somewhat true (2)
**COVID-19 RRR Survey**					
A1: Depressive Mood	NIH Sadness	felt_sad_cv	In the past week: I felt sad	1= Never; 2= Almost Never; 3= Sometimes; 4= Often; 5= Almost Always	Often (4) or above
		felt_always_sad	In the past week.: I could not stop feeling sad	1= Never; 2= Almost Never; 3= Sometimes; 4= Often; 5= Almost Always	Often (4) or above

© 2025 Gataviņš MM et al. *JAMA Network Open*.

		felt_unhappy_cv	In the past week.: I felt unhappy	1= Never; 2= Almost Never; 3= Sometimes; 4= Often; 5= Almost Always	Often (4) or above
		felt_angry_cv	In the past week.: I felt angry or frustrated	1= Never; 2= Almost Never; 3= Sometimes; 4= Often; 5= Almost Always	Almost always (5)
A2: Anhedonia		felt_no_fun_cv	It was hard for me to have fun	1= Never; 2= Almost Never; 3= Sometimes; 4= Often; 5= Almost Always	Often (4) or above

© 2025 Gataviņš MM et al. *JAMA Network Open*.

	NIH Positive Affect	interested_y_cv	Please rate how each item describes you now or within the past week. I felt interested	1 = Not true; 3 = Somewhat true; 5 = Very true	Not true (1)

© 2025 Gataviņš MM et al. *JAMA Network Open*.

A4: Sleep problems	MCTQ	mctq_fd_min_to_sleep_cv	Report on your typical sleep behavior over the past week.: I need __________ minutes to fall asleep.	0 = 0 ; 1 = 1 ; 2 = 2 ; 3 = 3 ; 4 = 4 ; 5 = 5 ; 6 = 6 ; 7 = 7 ; 8 = 8 ; 9 = 9 ; 10 = 10 ; 11 = 15 ; 12 = 20 ; 13 = 25 ; 14 = 30 ; 15 = 40 ; 16 = 50 ; 17 = 1 hour ; 18 = 1 hour 15 minute ; 19 = 1 hour 30 minute ; 20 = 1 hour 45 minute ; 21 = 2 hours ; 22 = 3 hours ; 23 = 4 hours	1 hour (17) or above

© 2025 Gataviņš MM et al. *JAMA Network Open*.

		mctq_fd_num_ wake_up_cv	Report on your typical sleep behavior over the past week.: After falling asleep, I wake up ____ times during the night.	0 = 0 ; 1 = 1 ; 2 = 2 ; 3 = 3 ; 4 = 4 ; 5 = 5 ; 6 = 6 ; 7 = 7 ; 8 = 8 ; 9 = 9 ; 10 = 10	2 or more times
A6: Fatigue	NIH Positive Affect	energetic_y_cv	Please rate how each item describes you now or within the past week. I felt energetic	1 = Not true; 3 = Somewhat true; 5 = Very true	Not true (1)
A7: Sense of guilt & [illegible]	NIH Sadness	felt_cv	In the past week.: I felt like I couldn't do anything right	1= Never; 2= Almost Never; 3= Sometimes; 4= Often; 5= Almost Always	Often (4) or above

© 2025 Gataviņš MM et al. *JAMA Network Open*.

		felt_life_went_wrong_cv	In the past week.: I felt everything in my life went wrong	1= Never; 2= Almost Never; 3= Sometimes; 4= Often; 5= Almost Always	Often (4) or above
A8: Attention and concentration problems	NIH Positive Affect	attentive_y_cv	Please rate how each item describes you now or within the past week. I felt attentive (that is, alert or able to pay attention)	1 = Not true; 3 = Somewhat true; 5 = Very true	Not true (1)
		concentrate_y_cv	Please rate how each item describes you now or within the past week. I felt able to concentrate	1 = Not true; 3 = Somewhat true; 5 = Very true	Not true (1)

© 2025 Gataviņš MM et al. *JAMA Network Open*.

eTable 3. Missingness of depression score across study timepoints

Symptom	Pre-pandemic	During pandemic				Post-pandemic
		Bin 1	Bin 2	Bin 3	Bin 4	
**Participants**	**3512**	**2799**	**2638**	**2399**	**1655**	**3512**
**A1**	49 (1.4%)	210 (7.5%)	197 (7.5%)	212 (8.8%)	93 (5.6%)	69 (2.0%)
**A2**	1452 (41.3%)	624 (22.3%)	387 (14.7%)	383 (16.0%)	101 (6.1%)	1341 (38.2%)
**A4**	1390 (39.6%)	27 (1.0%)	24 (0.9%)	39 (1.6%)	43 (2.6%)	14 (0.4%)
**A6**	1452 (41.3%)	897 (32.0%)	380 (14.4%)	376 (15.7%)	493 (29.8%)	1341 (38.2%)
**A7**	72 (2.1%)	213 (7.6%)	195 (7.4%)	216 (9.0%)	97 (5.9%)	73 (2.1%)
**A8**	63 (1.8%)	411 (14.7%)	192 (7.3%)	209 (8.7%)	502 (30.3%)	28 (0.8%)

The number of participants missing a score for each symptom across the six periods of interest (pre-, during, and post-pandemic). The missingness percentages are calculated from the number of participants with any data during the respective bin.

© 2025 Gataviņš MM et al. *JAMA Network Open*.

eTable 4. Latent growth mixed model fit statistics depressive symptoms' trajectories

**Fit Indices**	**1 class**	**2 classes**	**3 classes**	**4 classes**	**5 classes**
Akaike information criterion	42338.845	40731.593	**40208.041**	39735.936	39438.265
Bayesian information criterion	42400.484	40811.724	**40306.664**	39853.051	39573.872
Sample size-adjusted Bayesian information criterion	42368.709	40770.417	**40255.824**	39792.679	39503.967
Entropy	--	0.910	**0.921**	0.909	0.908
Lo-Mendell-Rubin adjusted likelihood ratio test, *P*	--	0.0059	**0.0652**	0.2228	0.6985

© 2025 Gataviņš MM et al. *JAMA Network Open*.

eTable 5. Intercept and slope statistics of the three-class trajectories model

		Intercept Factor			*Slope Factor*		
Depression Trajectory Class	*N*	B	SE	*p*-value	B	SE	*p*-value
**Resilient**	3027	.22	.07	.038	.04	.02	.128
**Susceptible**	326	-.05	.30	.875	.54	.08	.002
**Chronic**	159	2.31	.19	<.001	-.04	.05	.505

© 2025 Gataviņš MM et al. *JAMA Network Open*.

eTable 6. Comparisons of post-pandemic mental health diagnoses between resilient and depression-susceptible trajectories

Instrument	Characteristic	Resilient<br>N = 3,027	Susceptible<br>N = 326	P	Missing,<br>N (%)
K-SADS-Y	**Depression, n (%)**	**114 (6.2%)**	**123 (56.2%)**	**<0.001**	1288 (38.4%)
	**Anxiety, n (%)**	**117 (6.3%)**	**82 (37.6%)**	**<0.001**	1289 (38.4%)
	**Bipolar disorder, n (%)**	**32 (1.7%)**	**21 (9.6%)**	**<0.001**	
	**Sleep problems, n (%)**	**287 (15.6%)**	**113 (51.8%)**	**<0.001**	
K-SADS-P	Attention-deficit/hyperactivity disorder, n (%)	255 (8.4%)	33 (10.1%)	0.3	2 (<0.1%)
	Conduct disorder, n (%)	77 (2.5%)	14 (4.3%)	0.065	
	**Obsessive-compulsive disorder, n (%)**	**60 (3.2%)**	**16 (7.2%)**	**0.002**	1260 (37.6%)
	**Post-traumatic stress disorder, n (%)**	**50 (2.7%)**	**17 (7.7%)**	**<0.001**	
	Oppositional-defiant disorder, n (%)	100 (5.3%)	17 (7.7%)	0.15	1259 (37.5%)
	**Psychosis, n (%)**	**3 (<0.1%)**	**6 (1.8%)**	**<0.001**	2 (<0.1%)
BPM-Y	**Attention score, mean (SD)**	**3.4 (2.6)**	**6.3 (2.7)**	**<0.001**	630 (18.8%)
	**Internalizing score, mean (SD)**	**1.9 (2.1)**	**5.3 (3.1)**	**<0.001**	573 (17.1%)
	**Externalizing score, mean (SD)**	**1.9 (1.8)**	**3.5 (2.4)**	**<0.001**	612 (18.3%)
	**Total problem score, mean (SD)**	**7.0 (5.2)**	**14.9 (5.9)**	**<0.001**	727 (21.7%)

Mental health diagnosis and scores comparisons of resilient and susceptible youth. Data was taken from Kiddie Schedule for Affective Disorders (K-SADS) youth (-Y) and parent (-P) -report and Brief Problem Monitor for Youth (BPM-Y). The data was assessed at the post-pandemic follow-up. Diagnoses (all K-SADS measures) are reported as number and percentage of all non-missing values (n [%]); scores from BPM-Y are reported as mean (SD). For continuous variables, Kruskal-Wallis' test was used; for categorical – either Pearson's chi-square or Fisher's exact test (if either group had a count of below 5, the latter was used). Missingness is reported as number of participants missing a specific diagnosis score and as a percentage of participants missing the item from all resilient and susceptible class subjects (total N=3,353; N [%]).

© 2025 Gataviņš MM et al. *JAMA Network Open*.

eTable 7. Demographic comparison of resilient, susceptible, and chronic trajectory groups

Characteristic	Chronic (C) N = 159	Resilient (R) N = 3,027	Susceptible (S) N = 326	P	Significant post-hoc [P_{FDR}]
**Female sex, n (%)**	**105 (66.0%)**	**1,327 (43.9%)**	**240 (73.6%)**	**<0.001**	**R < C [<0.001]**<br>**R < S [<0.001]**
White, n (%)	114 (72.6%)	2,212 (73.2%)	235 (72.1%)	0.9	
Black, n (%)	43 (27.4%)	681 (22.5%)	75 (23.0%)	0.4	
Other race, n (%)	11 (7.0%)	187 (6.2%)	14 (4.3%)	0.3	
**Asian, n (%)**	**5 (3.2%)**	**200 (6.6%)**	**31 (9.5%)**	**0.027**	**C < S [0.040]**
**American Indian/Alaska Native**, n (%)	**14 (8.9%)**	**95 (3.1%)**	**12 (3.7%)**	**<0.001**	**R < C [<0.001]**<br>**S < C [0.025]**
Native Hawaiian/Other PI, n (%)	1 (0.6%)	15 (0.5%)	2 (0.6%)	0.6	
**Mixed race**, n (%)	**30 (19.1%)**	**356 (11.8%)**	**45 (13.8%)**	**0.016**	**R < C [0.018]**
**Parent education**, n (%)				**0.021**	
**Below high school**	**7 (4.4%)**	**160 (5.3%)**	**14 (4.3%)**		
**High school graduate**	**13 (8.2%)**	**279 (9.2%)**	**34 (10.5%)**		
**Some post-high school education**	**52 (32.7%)**	**718 (23.7%)**	**99 (30.6%)**		
**Bachelor's degree**	**44 (27.7%)**	**759 (25.1%)**	**77 (23.8%)**		
**Master's degree or above**	**43 (27.0%)**	**1,109 (36.7%)**	**100 (30.9%)**		
Below FPL, n (%)	29 (20.4%)	397 (14.5%)	50 (16.8%)	0.10	
Hispanic ethnicity, n (%)	37 (23.6%)	586 (19.6%)	74 (22.8%)	0.2	
Born in USA, n (%)	151 (95.0%)	2,923 (96.8%)	314 (96.6%)	0.5	
**Age in March 2020, mean (SD)**	**12.15 (0.75)**	**12.03 (0.86)**	**12.19 (0.82)**	**0.001**	**R < S [0.004]**
**Household income, n (%)**				**0.006**	
< $25,000	**27 (18.6%)**	**350 (12.5%)**	**41 (13.4%)**		**C ≠ R [0.017]**
$25,000 – 50,000	**27 (18.6%)**	**380 (13.6%)**	**45 (14.7%)**		
$50,000 – 100,000	**43 (29.7%)**	**705 (25.2%)**	**93 (30.4%)**		
$100,000 – 200,000	**33 (22.8%)**	**940 (33.6%)**	**95 (31.1%)**		
>$200,000	**15 (10.3%)**	**423 (15.1%)**	**32 (10.5%)**		

© 2025 Gataviņš MM et al. *JAMA Network Open*.

Demographic comparisons of resilient, chronic, and depression-susceptible youth. For continuous variables, one-way ANOVA was used; for categorical – either Pearson's chi-square or Fisher's exact test (if either group had a count of below 5, the latter was used). For post-hoc testing, Tukey's HSD test was used for continuous and pairwise Pearson's chi-square or Fisher exact test for categorical (same logic as for multivariate comparisons tests, count < 5 for Fisher's). P-values were FDR corrected. Only the significant results (after FDR correction) are presented. Percentages are calculated from the number of non-missing values. PI = Pacific Islander.

© 2025 Gataviņš MM et al. *JAMA Network Open*.

eTable 8. Associations of late/post pubertal stage (binary measure) with depressive symptom trajectory class

IVs	***ORs***	***CI***	***P***	***ORs***	***CI***	***P***	***ORs***	***CI***	***P***
(Intercept)	0.09	0.08 – 0.10	**<0.001**	0.05	0.04 – 0.06	**<0.001**	0.05	0.04 – 0.06	**<0.001**
Late/post puberty	2.41	1.82 – 3.19	**<0.001**	1.46	1.08 – 1.96	**0.014**	1.00	0.31 – 3.29	0.998
Female sex				3.18	2.42 – 4.19	**<0.001**	3.11	2.34 – 4.12	**<0.001**
Depression score pre-COVID				1.22	1.04 – 1.44	**0.013**	1.23	1.05 – 1.44	**0.012**
Late/post puberty x Female sex							1.50	0.44 – 5.11	0.518
N	3171			3168			3168		
Marginal R^2	0.027			0.111			0.110		
Conditional R^2	0.044			0.129			0.129		

All models are nested by family and site (accounting for the multi-level hierarchical nature of ABCD data). Binary variable indicating depression-susceptible (1) or resilient (0) class membership was used as the dependent variable. IVs = independent variables, OR = odds ratio, CI = 95% confidence interval.

© 2025 Gataviņš MM et al. *JAMA Network Open*.

eTable 9. Associations of pubertal stage (5- point Likert scale) with depressive symptom trajectory class

IVs	***ORs***	***CI***	***P***	***ORs***	***CI***	***P***	***ORs***	***CI***	***P***
(Intercept)	0.03	0.02 – 0.04	**<0.001**	0.03	0.02 – 0.04	**<0.001**	0.04	0.02 – 0.07	**<0.001**
Pubertal stage	1.69	1.48 – 1.93	**<0.001**	1.30	1.12 – 1.51	**<0.001**	1.11	0.85 – 1.45	0.459
Female sex				2.82	2.11 – 3.76	**<0.001**	1.58	0.67 – 3.70	0.294
Depression score pre-COVID				1.20	1.03 – 1.41	**0.022**	1.20	1.03 – 1.41	**0.023**
Pubertal stage x Female sex							1.26	0.91 – 1.74	0.158
N	3171			3168			3168		
Marginal R^2	0.068			0.121			0.114		
Conditional R^2	0.080			0.135			0.129		

All models are nested by family and site (accounting for the multi-level hierarchical nature of ABCD data). Binary variable indicating depression-susceptible (1) or resilient (0) class membership was used as the dependent variable. IVs = independent variables, OR = odds ratio, CI = 95% confidence interval.

© 2025 Gataviņš MM et al. *JAMA Network Open*.

eTable 10. Depression trajectory models stratified by pubertal stage throughout the pandemic

Late/post-pubertal throughout the pandemic (N=486)							
		Intercept Factor			*Slope Factor*		
Depression Trajectory Class	*N*	B	SE	*p*-value	B	SE	*p*-value
**Resilient**	386	.36	.21	.165	.05	.05	.398
**Susceptible**	71	-.10	.24	.700	.53	.06	.001
**Chronic**	29	2.50	.20	<.001	.11	.05	.101
Pre/early/mid-pubertal stage throughout the pandemic (N=1,547)							
		Intercept Factor			*Slope Factor*		
**Resilient**	1306	.19	.05	.024	.02	.01	.272
**Susceptible**	178	.75	.09	.001	.16	.02	.002
**Chronic**	31	1.81	.51	.023	.22	.13	.161

© 2025 Gatavinš MM et al. *JAMA Network Open*.

eTable 11. Associations of pre-pandemic prosocial behaviors and problem-solving skills with depressive symptom trajectory class

IVs	*ORs*	*CI*	*P*	*ORs*	*CI*	*P*
(Intercept)	0	0.00 – 0.02	**<0.001**	0	0.00 – 0.00	**<0.001**
Female sex	3.68	2.83 – 4.78	**<0.001**	3.56	2.33 – 5.44	**<0.001**
Age in March 2020	1.24	1.08 – 1.43	**0.002**	1.57	1.25 – 1.96	**<0.001**
Depression score pre-COVID	1.27	1.09 – 1.48	**0.003**	1.30	0.82 – 2.08	0.265
Prosocial behaviors	0.97	0.86 – 1.10	0.665			
Problem-solving skills				0.80	0.66 – 0.97	**0.021**
N	3344			1384		
Marginal R^2	0.121			0.135		
Conditional R^2	0.167			0.223		

All models are nested by family and site (accounting for the multi-level hierarchical nature of ABCD data). Binary variable indicating depression-susceptible (1) or resilient (0) class membership was used as the dependent variable. IVs = independent variables, OR = odds ratio, CI = 95% confidence interval.

© 2025 Gataviņš MM et al. *JAMA Network Open*.

eTable 12. Associations of family environment with depressive symptom trajectory class

IVs	***ORs***	***CI***	***P***	***ORs***	***CI***	***P***
(Intercept)	0	0.00 – 0.01	**<0.001**	0	0.00 – 0.01	**<0.001**
Female sex	3.75	2.89 – 4.87	**<0.001**	3.92	3.01 – 5.11	**<0.001**
Age in March 2020	1.27	1.10 – 1.46	**0.001**	1.26	1.10 – 1.45	**0.001**
Depression score pre-COVID	1.20	1.02 – 1.40	**0.026**	1.21	1.03 – 1.41	**0.02**
Family conflict	1.23	1.10 – 1.38	**<0.001**			
Parental monitoring				0.81	0.73 – 0.91	**<0.001**
N	3348					
Marginal R^2	0.131			0.131		
Conditional R^2	0.175			0.178		

All models are nested by family and site (accounting for the multi-level hierarchical nature of ABCD data). Binary variable indicating depression-susceptible (1) or resilient (0) class membership was used as the dependent variable. IVs = independent variables, OR = odds ratio, CI = 95% confidence interval.

© 2025 Gataviņš MM et al. *JAMA Network Open*.

eTable 13. Associations of peer-victimization measures with depressive symptom trajectory class

IVs	***ORs***	***CI***	***P***	***ORs***	***CI***	***P***	***ORs***	***CI***	***P***
(Intercept)	0.01	0.00 – 0.11	**<0.001**	0.01	0.00 – 0.11	**<0.001**	0.01	0.00 – 0.11	**<0.001**
Female sex	3.48	2.52 – 4.81	**<0.001**	3.51	2.54 – 4.84	**<0.001**	3.55	2.57 – 4.92	**<0.001**
Age in March 2020	1.16	0.93 – 1.43	0.181	1.15	0.93 – 1.42	0.195	1.15	0.93 – 1.42	0.205
Depression score pre-COVID	1.24	1.04 – 1.46	**0.014**	1.23	1.04 – 1.46	**0.017**	1.20	1.01 – 1.43	**0.039**
Cyberbullying pre-COVID	2.28	1.45 – 3.59	**<0.001**				2.14	1.35 – 3.39	**0.001**
Victimization pre-COVID				1.71	1.11 – 2.65	**0.016**	1.52	0.97 – 2.39	0.068
N	2003			2008			2003		
Marginal R^2	0.123			0.116			0.127		
Conditional R^2	0.165			0.159			0.168		

All models are nested by family and site (accounting for the multi-level hierarchical nature of ABCD data). Binary variable indicating depression-susceptible (1) or resilient (0) class membership was used as the dependent variable. Binary variable indicating depression-susceptible (1) or resilient (0) class membership was used as the dependent variable. IVs = independent variables, OR = odds ratio, CI = 95% confidence interval.

© 2025 Gataviņš MM et al. *JAMA Network Open*.

eTable 14. Associations of parental history of depression with depressive symptom trajectory class

IVs	***ORs***	***CI***	***P***	***ORs***	***CI***	***P***
(Intercept)	0	0.00 – 0.02	**<0.001**	0	0.00 – 0.02	**<0.001**
Female sex	3.69	2.83 – 4.82	**<0.001**	3.87	2.94 – 5.09	**<0.001**
Age in March 2020	1.23	1.07 – 1.42	**0.004**	1.25	1.08 – 1.44	**0.003**
Depression score pre-COVID	1.27	1.09 – 1.49	**0.003**	1.31	1.12 – 1.53	**0.001**
Maternal history of depression	1.52	1.16 – 1.99	**0.002**			
Paternal history of depression				1.36	0.97 – 1.92	0.073
N	3196			3114		
Marginal R^2	0.129			0.132		
Conditional R^2	0.181			0.181		

All models are nested by family and site (accounting for the multi-level hierarchical nature of ABCD data). Binary variable indicating depression-susceptible (1) or resilient (0) class membership was used as the dependent variable. Binary variable indicating depression-susceptible (1) or resilient (0) class membership was used as the dependent variable. IVs = independent variables, OR = odds ratio, CI = 95% confidence interval.

© 2025 Gataviņš MM et al. *JAMA Network Open*.

eTable 15. Associations of pre-pandemic poly-environmental (exposome) adversity score and SES measures with depressive symptom trajectory class

IVs	*ORs*	*CI*	*P*	*ORs*	*CI*	*P*	*ORs*	*CI*	*P*	*ORs*	*CI*	*P*
(Intercept)	0.01	0.00 – 0.03	**<0.001**	0	0.00 – 0.03	**<0.001**	0	0.00 – 0.01	**<0.001**	0.01	0.00 – 0.10	**<0.001**
Female sex	3.56	2.72 – 4.65	**<0.001**	3.75	2.87 – 4.89	**<0.001**	3.64	2.80 – 4.73	**<0.001**	3.60	2.73 – 4.74	**<0.001**
Age in March 2020	1.23	1.06 – 1.42	**0.006**	1.21	1.05 – 1.39	**0.009**	1.3	1.13 – 1.50	**<0.001**	1.24	1.06 – 1.44	**0.006**
Depression score pre-COVID	1.24	1.05 – 1.45	**0.009**	1.28	1.10 – 1.50	**0.002**	1.19	1.02 – 1.40	**0.030**	1.20	1.02 – 1.42	**0.032**
Household income	0.96	0.91 – 1.01	0.094							1.04	0.97 – 1.11	0.289
ADI				1.00	0.99 – 1.01	0.919				0.98	0.98 – 0.99	**<0.001**
Pre-pandemic adverse exposome							1.28	1.13 – 1.45	**<0.001**	1.75	1.42 – 2.16	**<0.001**
N	3100			3116			3286			2851		
Marginal R^2	0.118			0.123			0.135			0.152		
Conditional R^2	0.167			0.173			0.182			-		

All models are nested by family and site (accounting for the multi-level hierarchical nature of ABCD data). Binary variable indicating depression-susceptible (1) or resilient (0) class membership was used as the dependent variable. IVs = independent variables, OR = odds ratio, CI = 95% confidence interval.

© 2025 Gataviņš MM et al. *JAMA Network Open*.

eTable 16. Associations of polygenic risk of depression with depressive symptom trajectories among European-like or African-like genetic ancestry youth

	European-like ancestry (EUR)			***African-like ancestry (AFR)***		
IVs	***ORs***	***CI***	***P***	***ORs***	***CI***	***P***
(Intercept)	0	0.00 – 0.03	**<0.001**	0.01	0.00 – 1.56	0.075
Age	1.32	1.06 – 1.65	**0.015**	1.11	0.74 – 1.67	0.601
Female sex	3.01	2.05 – 4.43	**<0.001**	3.32	1.62 – 6.79	**0.001**
Depression score pre-COVID	1.29	1.01 – 1.65	**0.042**	1.16	0.83 – 1.62	0.387
Depression polygenic risk	1.28	1.03 – 1.59	**0.023**	0.8	0.58 – 1.10	0.163
N	1525			466		
Marginal R^2	0.118			0.116		

All models are nested by family and site (accounting for the multi-level hierarchical nature of ABCD data). Binary variable indicating depression-susceptible (1) or resilient (0) class membership was used as the dependent variable. The depression polygenic risk score row corresponds to the polygenic risk score used for respective genetic ancestry participants (i.e., depression polygenic risk score values for the EUR column correspond to the depression polygenic risk score calculated from GWAS data of European-like ancestry participants and AFR column values to depression polygenic risk score from African-like ancestry participant GWAS data; see **eMethods 2** for calculation of polygenic risk scores). IVs = independent variables, OR = odds ratio, CI = 95% confidence interval.

© 2025 Gataviņš MM et al. *JAMA Network Open*.

eTable 17. Main and interactive associations of exposomic risk and polygenic risk of depression with depressive symptom trajectories among European-like ancestry youth

	**Model 1 (G only)**			**Model 2 (E only)**			**Model 3 (G + E)**			**Model 4 (G x E)**		
IVs	***ORs***	***CI***	***P***	***ORs***	***CI***	***P***	***ORs***	***CI***	***P***	***ORs***	***CI***	***P***
(Intercept)	0	0.00 – 0.02	**<0.001**	0	0.00 – 0.01	**<0.001**	0	0.00 – 0.02	**<0.001**	0	0.00 – 0.02	**<0.001**
Age	1.34	1.07 – 1.68	**0.011**	1.4	1.12 – 1.76	**0.004**	1.39	1.11 – 1.75	**0.005**	1.39	1.11 – 1.75	**0.005**
Female sex	2.92	1.98 – 4.30	**<0.001**	2.99	2.03 – 4.41	**<0.001**	2.97	2.01 – 4.38	**<0.001**	3.00	2.03 – 4.43	**<0.001**
Depression score pre-COVID	1.31	1.02 – 1.67	**0.034**	1.22	0.95 – 1.57	0.123	1.21	0.94 – 1.56	0.139	1.2	0.93 – 1.55	0.154
Exposome				1.52	1.22 – 1.89	**<0.001**	1.47	1.18 – 1.83	**0.001**	1.58	1.26 – 1.99	**<0.001**
Depression polygenic risk	1.28	1.03 – 1.58	**0.027**				1.21	0.98 – 1.51	0.083	1.15	0.92 – 1.43	0.223
Exposome x depression polygenic risk										0.72	0.56 – 0.93	**0.013**
N	1510											
Marginal R^2	0.115			0.126			0.139			0.151		

All models are nested by family and site (accounting for the multi-level hierarchical nature of ABCD data). The sample of 1510 European-like ancestry participants include those that have both exposome score and polygenic risk score data.

© 2025 Gataviņš MM et al. *JAMA Network Open*.

Binary variable indicating depression-susceptible (1) or resilient (0) class membership was used as the dependent variable. IVs = independent variables, OR = odds ratio, CI = 95% confidence interval.

χ^2 test for model fit comparison
Model 1 vs 3 (addition of E to G): **P = .001*****
Model 2 vs 3 (addition of G to E): P = .073

© 2025 Gataviņš MM et al. *JAMA Network Open*.

eTable 18. Main and interactive associations of exposomic risk and polygenic risk of depression with depressive symptom trajectory class among African-like ancestry youth

	**Model 1 (G only)**			**Model 2 (E only)**			**Model 3 (G + E)**			**Model 4 (G x E)**		
IVs	***ORs***	***CI***	***P***	***ORs***	***CI***	***P***	***ORs***	***CI***	***P***	***ORs***	***CI***	***P***
(Intercept)	0.01	0.00 – 1.18	0.058	0	0.00 – 0.63	**0.033**	0	0.00 – 0.74	**0.038**	0	0.00 – 0.75	**0.039**
Age	1.15	0.76 – 1.75	0.504	1.22	0.80 – 1.87	0.36	1.20	0.78 – 1.85	0.396	1.20	0.78 – 1.85	0.396
Female sex	3.50	1.66 – 7.37	**0.001**	3.41	1.62 – 7.16	**0.001**	3.53	1.67 – 7.43	**0.001**	3.53	1.67 – 7.47	**0.001**
Depression score pre-COVID	1.12	0.79 – 1.59	0.515	1.11	0.78 – 1.58	0.547	1.10	0.77 – 1.57	0.594	1.10	0.77 – 1.57	0.598
Exposome				1.24	0.85 – 1.82	0.262	1.21	0.83 – 1.77	0.320	1.21	0.82 – 1.81	0.338
Depression polygenic risk	0.78	0.57 – 1.08	0.139				0.79	0.57 – 1.10	0.167	0.79	0.48 – 1.31	0.357
Exposome x depression polygenic risk										1.01	0.71 – 1.42	0.968
N	443											
Marginal R^2	0.124			0.118			0.129			0.130		

All models are nested by family and site (accounting for the multi-level hierarchical nature of ABCD data). The sample of 443 African-like ancestry participants includes those that have both exposome score and polygenic risk score data. Binary variable indicating depression-susceptible (1) or resilient (0) class membership was used as the dependent variable. IVs = independent variables, OR = odds ratio, CI = 95% confidence interval.

© 2025 Gataviņš MM et al. *JAMA Network Open*.

eTable 19. Trajectory model's intercept and slope statistics when using sensitive criteria of depressive symptoms

		Intercept Factor			*Slope Factor*		
Depression Trajectory Class	*N*	B	SE	*p*-value	B	SE	*p*-value
**Resilient**	2667	.69	.33	.103	-.00	.08	.971
**Susceptible**	646	1.12	.09	<.001	.24	.02	.001
**Chronic**	199	2.30	.31	.002	.33	.08	.015

© 2025 Gataviņš MM et al. *JAMA Network Open*.

eTable 20. Trajectory model's intercept and slope statistics when using imputed symptom scores

		Intercept Factor			*Slope Factor*		
Depression Trajectory Class	*N*	B	SE	*p*-value	B	SE	*p*-value
**Resilient**	3070	.40	.23	.159	.04	.06	.587
**Susceptible**	285	.43	.37	.310	.49	.09	.007
**Chronic**	157	2.42	.28	.001	-.12	.07	.186

© 2025 Gataviņš MM et al. *JAMA Network Open*.

eFigure 1. Timeline of COVID-19 pandemic events during data collection period

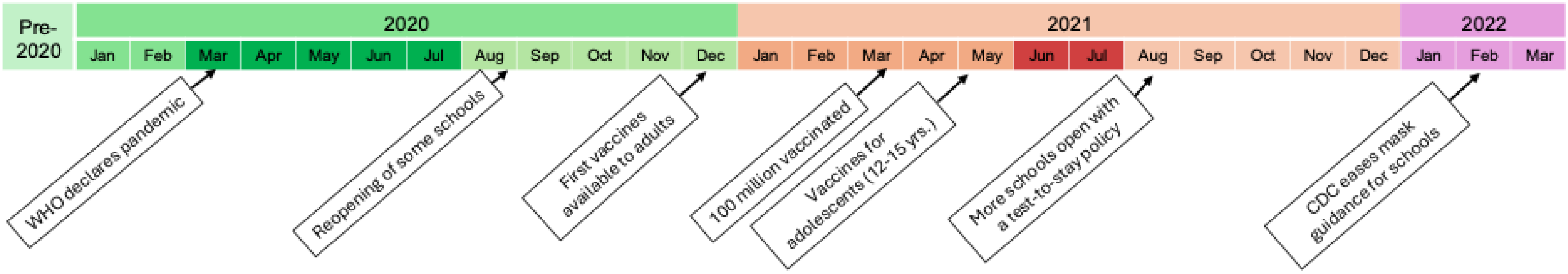

© 2025 Gataviņš MM et al. *JAMA Network Open*.

eFigure 2. Puberty-related differences between resilient and depression-susceptible trajectory classes

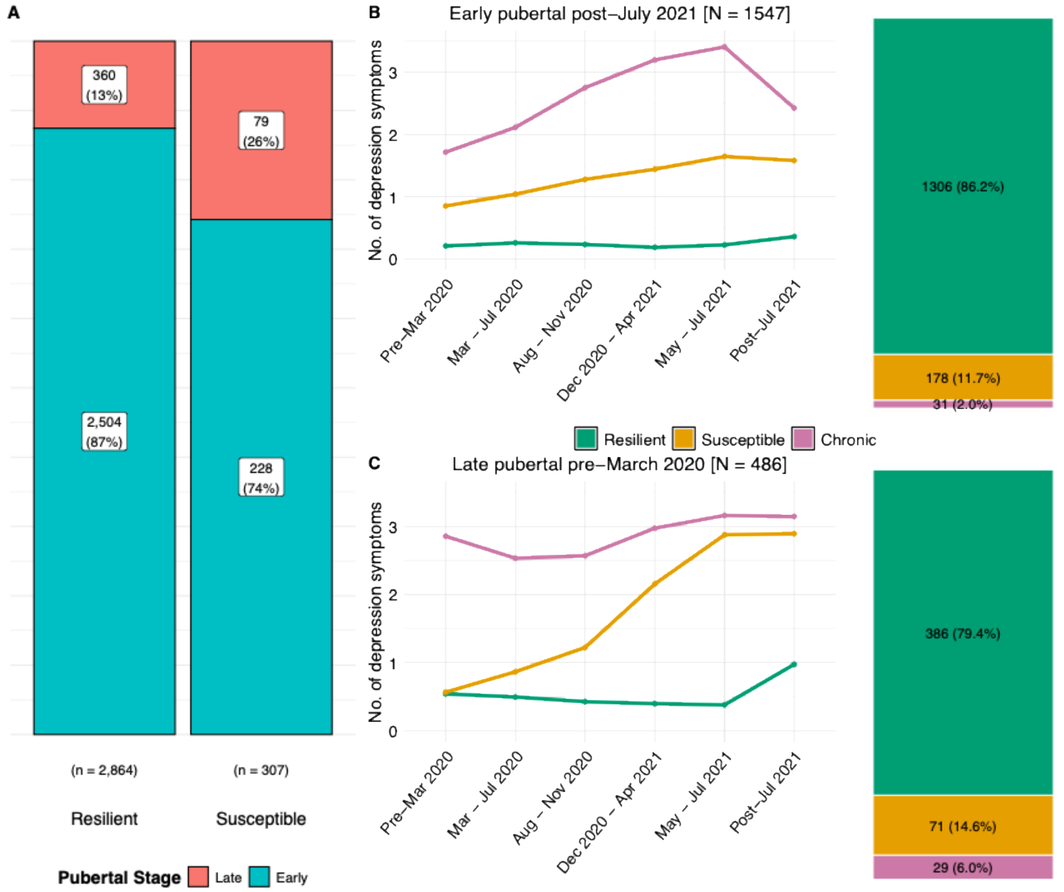

(A) Stacked bar chart comparing the number of participants in late and post-pubertal stages (denoted 'late') and early puberty pre-pandemic. (B) Three-class solution for depressive symptom trajectories of participants who were in late puberty pre-pandemic shows a significant depression-susceptible class slope, suggesting higher susceptibility to depression in those entering the pandemic in later puberty. (C) Three-class solution for depressive symptom trajectories of participants who were in early stages even post-pandemic (July 2021). Susceptible class slope is significant, but with a lower slope coefficient, suggesting a possibly lower susceptibility in those entering the pandemic in early puberty.

© 2025 Gataviņš MM et al. *JAMA Network Open*.

eFigure 3. Visualization depressive symptom scores trajectories using sensitive criteria of depressive symptoms

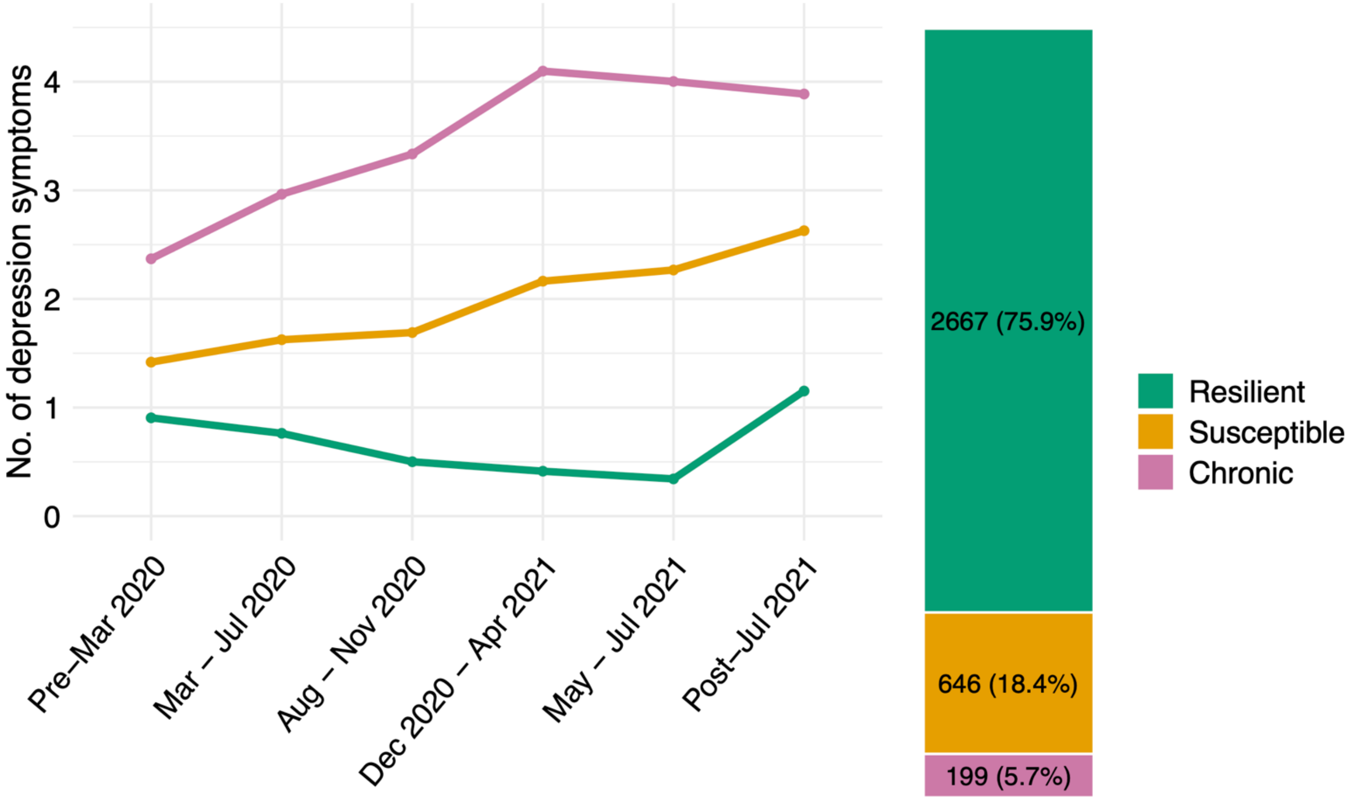

© 2025 Gataviņš MM et al. *JAMA Network Open*.

eFigure 4. Visualization depressive symptom scores trajectories using imputed symptom scores

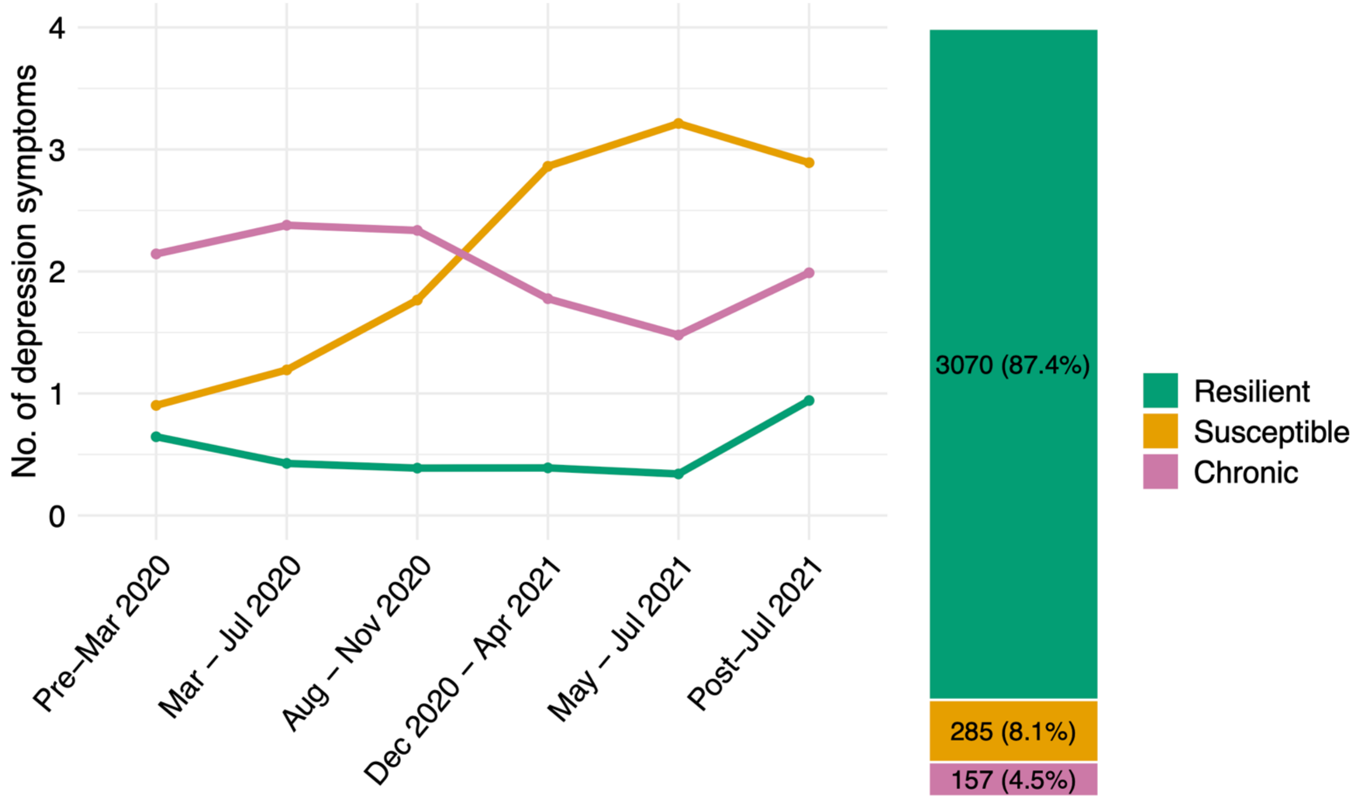

© 2025 Gataviņš MM et al. *JAMA Network Open*.

eFigure 5. Multi-level prospectively measured pre-pandemic risk and resilience factors, when adjusting for parent-reported family conflict during the pandemic

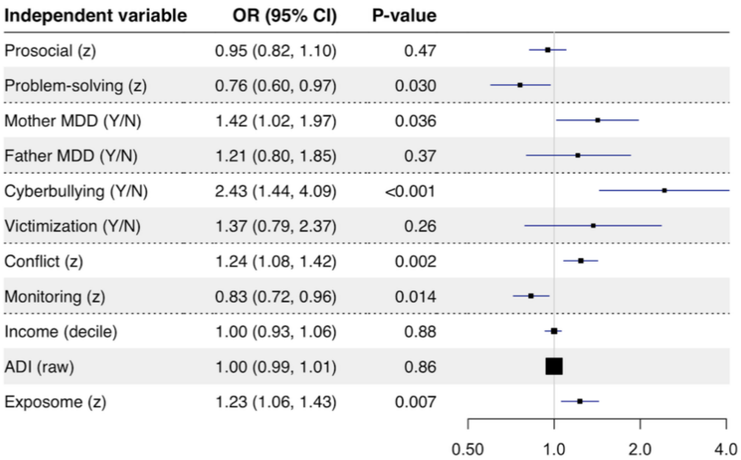

Independent variable	OR (95% CI)	P-value
Prosocial (z)	0.95 (0.82, 1.10)	0.47
Problem-solving (z)	0.76 (0.60, 0.97)	0.030
Mother MDD (Y/N)	1.42 (1.02, 1.97)	0.036
Father MDD (Y/N)	1.21 (0.80, 1.85)	0.37
Cyberbullying (Y/N)	2.43 (1.44, 4.09)	<0.001
Victimization (Y/N)	1.37 (0.79, 2.37)	0.26
Conflict (z)	1.24 (1.08, 1.42)	0.002
Monitoring (z)	0.83 (0.72, 0.96)	0.014
Income (decile)	1.00 (0.93, 1.06)	0.88
ADI (raw)	1.00 (0.99, 1.01)	0.86
Exposome (z)	1.23 (1.06, 1.43)	0.007

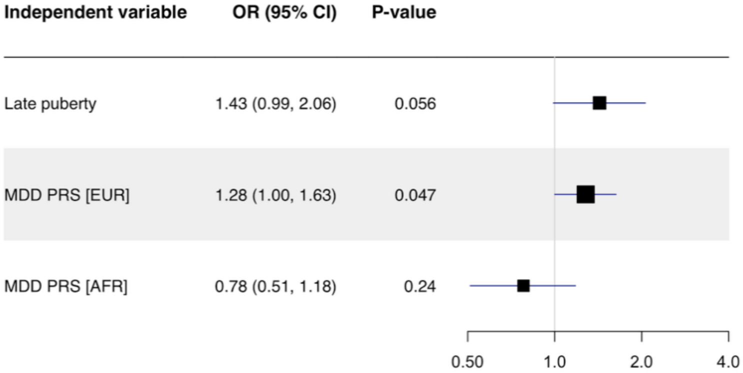

Independent variable	OR (95% CI)	P-value
Late puberty	1.43 (0.99, 2.06)	0.056
MDD PRS [EUR]	1.28 (1.00, 1.63)	0.047
MDD PRS [AFR]	0.78 (0.51, 1.18)	0.24

We performed logistic mixed effects regression modeling using binary dependent variables (resilient = 0 and depression-susceptible = 1) and a set of measures from pre-pandemic assessments, including mean parent-report, pandemic-time ((between March 2020 and July 2021) increase in family conflict as a covariate in the models. Odds ratio (OR) > 1 indicates that the factor is linked to risk more than resilience; an OR < 1 indicates the factor is more linked to resilience than risk. Whiskers in the OR forest plots denote 95% confidence intervals. All p-values used to denote significance were uncorrected. (Above) Environmental measures of risk and resilience. Odds ratio axis is base-10 logarithmic (log10) scaled. (Below) Biological measures of risk and resilience. Odds ratio axis is plotted using log10 scaling. Legend: z = z-scored measure, Y/N = yes/no binary measure, MDD = Major Depressive Disorder, PRS = depression polygenic risk score, EUR = European-like genetic ancestry, AFR = African-like genetic ancestry, ADI = Area Deprivation Index, SD = standard deviation, CI = confidence interval.

© 2025 Gataviņš MM et al. *JAMA Network Open*.

eFigure 6. Multi-level prospectively measured pre-pandemic risk and resilience factors, when adjusting for parent-reported financial strain during the pandemic

Independent variable	OR (95% CI)	P-value
Prosocial (z)	0.95 (0.83, 1.08)	0.43
Problem-solving (z)	0.74 (0.59, 0.92)	0.008
Mother MDD (Y/N)	1.41 (1.03, 1.92)	0.031
Father MDD (Y/N)	1.17 (0.78, 1.75)	0.45
Cyberbullying (Y/N)	2.44 (1.48, 4.03)	<0.001
Victimization (Y/N)	1.39 (0.83, 2.34)	0.21
Conflict (z)	1.20 (1.06, 1.37)	0.005
Monitoring (z)	0.82 (0.72, 0.94)	0.004
Income (decile)	1.00 (0.93, 1.07)	0.93
ADI (raw)	1.00 (0.99, 1.00)	0.64
Exposome (z)	1.25 (1.07, 1.46)	0.004

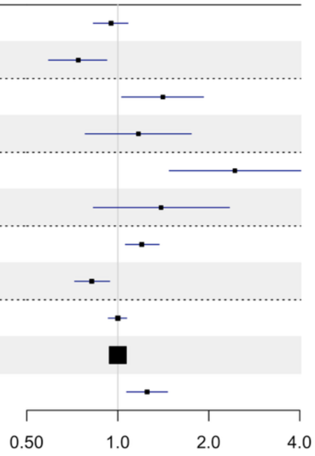

Independent variable	OR (95% CI)	P-value
Late puberty	1.64 (1.17, 2.30)	0.004
MDD PRS [EUR]	1.25 (0.99, 1.58)	0.066
MDD PRS [AFR]	0.78 (0.54, 1.13)	0.18

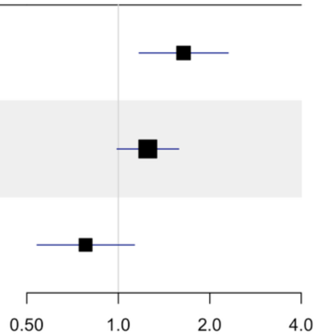

We performed logistic mixed effects regression modeling using binary dependent variables (resilient = 0 and depression-susceptible = 1) and a set of measures from pre-pandemic assessments, including mean parent-report, pandemic-time (between March 2020 and July 2021) financial strain as a covariate in the models. Odds ratio (OR) > 1 indicates that the factor is linked to risk more than resilience; an OR < 1 indicates the factor is more linked to resilience than risk. Whiskers in the OR forest plots denote 95% confidence intervals. All p-values used to denote significance were uncorrected. (Above) Environmental measures of risk and resilience. Odds ratio axis is base-10 logarithmic (log10) scaled. (Below) Biological measures of risk and resilience. Odds ratio axis is plotted using log10 scaling. Legend: z = z-scored measure, Y/N = yes/no binary measure, MDD = Major Depressive Disorder, PRS = depression polygenic risk score, EUR = European-like genetic ancestry, AFR = African-like genetic ancestry, ADI = Area Deprivation Index, SD = standard deviation, CI = confidence interval.

© 2025 Gataviņš MM et al. *JAMA Network Open*.
