# Supplementary material for: Adolescent Depressive Symptom Trajectories From Before to After the COVID-19 Pandemic
Source: JAMA Netw Open. 2025 Dec 1;8(12):e2545987. doi: 10.1001/jamanetworkopen.2025.45987 (PMC12670201; doi:10.1001/jamanetworkopen.2025.45987)
Supplement: Supplement 2. — Data Sharing Statement [file jamanetwopen-e2545987-s002.pdf]

Data Sharing Statement

Gataviņš. Adolescent Depressive Symptom Trajectories From Before to After the COVID-19 Pandemic. *JAMA Netw Open*. Published December 01, 2025.
doi:10.1001/jamanetworkopen.2025.45987

Data
Data available: No

Additional Information
Explanation for why data not available: Data used in the preparation of this article were obtained from the Adolescent Brain Cognitive Development Study (https://abcdstudy.org), held in the National Institute of Mental Health Data Archive and available for researchers upon application (https://nda.nih.gov/abcd/request-access). All code used for analyses is available at https://github.com/barzilab1/ABCD_Psych_Resilience.
